# Cross-institutional outcome prediction for head and neck cancer patients using self-attention neural networks

**DOI:** 10.1038/s41598-022-07034-5

**Published:** 2022-02-24

**Authors:** William Trung Le, Eugene Vorontsov, Francisco Perdigón Romero, Lotfi Seddik, Mohamed Mortada Elsharief, Phuc Felix Nguyen-Tan, David Roberge, Houda Bahig, Samuel Kadoury

**Affiliations:** 1 Polytechnique Montréal, 500 Chemin de Polytechnique, Montreal, QC H3T 1J4 Canada; 2 Centre de recherche du Centre Hospitalier de l’Université de Montréal, 900 Rue Saint-Denis, Pavillon R, Montreal, QC H2X 0A9 Canada; 3 Centre Hospitalier de l’Université de Montréal, 1051 Rue Sanguinet, Montreal, QC H2X 3E4 Canada

**Keywords:** Cancer imaging, Predictive markers, Computational science, Scientific data

## Abstract

In radiation oncology, predicting patient risk stratification allows specialization of therapy intensification as well as selecting between systemic and regional treatments, all of which helps to improve patient outcome and quality of life. Deep learning offers an advantage over traditional radiomics for medical image processing by learning salient features from training data originating from multiple datasets. However, while their large capacity allows to combine high-level medical imaging data for outcome prediction, they lack generalization to be used across institutions. In this work, a pseudo-volumetric convolutional neural network with a deep preprocessor module and self-attention (PreSANet) is proposed for the prediction of distant metastasis, locoregional recurrence, and overall survival occurrence probabilities within the 10 year follow-up time frame for head and neck cancer patients with squamous cell carcinoma. The model is capable of processing multi-modal inputs of variable scan length, as well as integrating patient data in the prediction model. These proposed architectural features and additional modalities all serve to extract additional information from the available data when availability to additional samples is limited. This model was trained on the public Cancer Imaging Archive Head–Neck-PET–CT dataset consisting of 298 patients undergoing curative radio/chemo-radiotherapy and acquired from 4 different institutions. The model was further validated on an internal retrospective dataset with 371 patients acquired from one of the institutions in the training dataset. An extensive set of ablation experiments were performed to test the utility of the proposed model characteristics, achieving an AUROC of $80\%$, $80\%$ and $82\%$ for DM, LR and OS respectively on the public TCIA Head–Neck-PET–CT dataset. External validation was performed on a retrospective dataset with 371 patients, achieving $69\%$ AUROC in all outcomes. To test for model generalization across sites, a validation scheme consisting of single site-holdout and cross-validation combining both datasets was used. The mean accuracy across 4 institutions obtained was $72\%$, $70\%$ and $71\%$ for DM, LR and OS respectively. The proposed model demonstrates an effective method for tumor outcome prediction for multi-site, multi-modal combining both volumetric data and structured patient clinical data.

## Introduction

Radiation therapy as a primary cancer treatment method has gained significant importance within the last few decades and is now used to treat approximately 50% of all cancer cases^1–3^ and 74% of head and neck (H&N) cancer cases^4^. In medical imaging, tumor characterization to stratify risk has been at the forefront of research goals in order to help improve prognosis^5–13^, including lowering the incidence of the following outcomes after treatment: locoregional recurrence (LR), distant metastasis (DM) and a second primary cancer following radiotherapy treatment. Both of these cancer progression outcomes can decrease probability of overall patient survival (OS). Furthermore, in selected H&N cancers, DM can be fatal to 50% of patients^14^, despite radiotherapy controlling the original locoregional disease in 90% of cases^14^. It thus remains imperative that early assessment of the tumor risk be made available to clinicians, as this can help physicians stratify patients by risk categories, guiding their choice intensify or deintensify the treatment according to risk stratification, as well as deciding the amount of systemic versus locoregional therapy afforded to the patient.

Within the last decade, deep learning methods have seen an explosive surge in popularity and have proven effective for outcome prediction tasks, especially those focusing on training data sources with high dimensionality such as text^15,16^, images^17,18^, videos^19^ and time-series^20^. In medical imaging, convolutional neural networks (CNN) have become the preferred approach for several radiological tasks^21^. Unlike physicians who make assessments based on visually discernible features from the imaging data while taking into account demographic parameters, CNNs learn a hierarchical composition of features from training data in a completely automated manner. In the past decade, neural network approaches have gained traction in classification, segmentation^22^, registration^23^, domain translation tasks for treatment planning^24^, image-guided interventions, and patient follow-up care^21,25^.

The recent success of deep neural networks (DNN) has been driven in part by the availability of large training datasets^26^. Indeed DNN models see performance improvement scaling with the size the training data. For this reason, the benchmark 14 million samples ImageNet dataset^27^ has seen performance from CNNs exceeding human levels at classification tasks. While this remains impressive in a benchmarking setting, the reality is different in the medical imaging field. Due to the difficulty in acquiring patient scans for research, training data in medical imaging is much more scarce. Moreover, on top of the different modalities available to capture the same anatomy with all of the information trade offs they provide such as X-ray computed tomography (CT) and positron emission tomography (PET), scans will often present difficulty to discern differences due to conditions between institutions that are not reproducible; scanner manufacturer and models, reconstruction algorithm choice, and operator handling may all contribute to this effect. All of these variabilities compound the difficulty of modeling classification tasks on small training samples. For these reasons, it is important to assess the generalizability of deep learning models across institutions.

This work builds on the 2017 “Head–Neck-PET–CT”^28^ dataset, released as part of the Cancer Imaging Archive (TCIA)^29^, for multi-institution outcome prediction of head and neck cancer cases. This dataset contained 298 cases of H&N cancer undergoing radiation therapy or chemoradiation therapy. Each patient data included fluorodeoxyglucose (FDG) PET–CT image data along with segmentation annotations and clinical data. This dataset is also useful to benchmark multi-domain medical imaging processing tasks, given that it contains patient data from 4 different institutions in Québec Canada: the Centre Hospitalier de l’Université de Montréal (CHUM), the Centre Hospitalier Universitaire de Sherbrooke (CHUS), the Jewish General Hospital (HGJ) and the Maisonneuve-Rosemont Hospital (HMR). Initial work used a radiomics approach combined with a random forest classifier for tumor risk assessment^30^. Methods exploited the multi-modal nature of the dataset by incorporating features from both PET and CT volumes, as well as clinical data coming from patient demographics. Diamant et al.^31^ explored the use of deep learning techniques for H&N cancer outcome predictions, using a CNN model. Their custom-made CNN was composed of 3 convolutional and 2 fully-connected layers, a considerably simpler architecture than modern CNN approaches for image processing^32^. Compared with the previous random forest approach trained and validated on the same dataset, it was limited to using CT only, exploiting a single slice containing the largest gross tumor volume (GTV) surface area. Furthermore, the image itself was masked to contain only information within the tumor contour.

We propose PreSANet (pseudo-volumetric neural network with deep preprocessor module and self-attention layers), an end-to-end trainable framework for H&N PET–CT classification of radio/chemo-radiotherapy treatment response using a multi-branch CNN taking both medical images and clinical data as inputs. The method is designed to extract as much information from the available dataset as possible via various state-of-the-art deep learning techniques. Both CT and PET data are processed in parallel as separate channels at the input of the model, allowing complementary structural and functional information to be integrated during the learning process. To exploit the three-dimensional nature of the images, a novel pseudo-volumetric technique is used in which a weight-shared convolutional sub-module is used on each slice before having all latent mapping being combined into a single representation via a pair of length independent statistics. Based on the works of Havaei et al.^33^, our method is a trade-off between the computationally expensive full 3D and the lossy 2D CNNs architectures. Furthermore, it presents a unique way of making the model robust to the variable depth nature of medical images, for which bounding boxes or the random patch technique are traditionally used. Compared with previous CNN based approaches, the proposed model incorporates various state-of-the-art deep learning techniques to account for the inter-patient and inter-institutional variabilities. To normalize differences in input images across multiple institutions in the training dataset, a deep convolutional image-to-image preprocessor sub-module based on the work of Drozdzal et al.^34^ is integrated end-to-end with the prediction network. Previous work demonstrated that the initial fully-convolutional network (FCN) component of their segmentation model acted as a domain normalizer on medical images, yielding state-of-the-art performance on liver lesion segmentation. We hypothesize that the inclusion of this FCN as a first component of the classification approach would also yield improved predictive performance on a multi-institutional dataset. Following this, the building blocks of the CNN are augmented with aggregated residual connections, a technique that allows passing high-level features to downstream layers^35^, which was shown to improve convergence by preventing the vanishing gradient problem. Each convolutional block is succeeded by a global context (GC) block based on the work of Cao et al.^36^. This self-attention mechanism allows the model to spatially re-focus on the salient inter-patient features extracted in the previous step.

The aim of this work is to demonstrate the predictive capability of the PreSANet architecture on both a public and internal datasets for tumor outcome prediction. Furthermore, we demonstrate the generalization capabilities of PreSANet by validating across both available datasets. The novel contributions are fourfold, and are presented as a series of three different categories of experiments:

- We evaluate the performance gains of the various components of the proposed model in an ablation study. These include the preprocessor module, the self-attention layers and the pseudo-volumetric approach based on HeMIS^33^, as well as the addition of paired PET images and clinical data to supplement the planning CT input.
- A comparison in accuracy is performed on the “Head–Neck-PET–CT”^28^ public dataset and compare it previous approaches trained with the dataset^30,31^, as well as those by off-the-shelf CNN models^37^ and a traditional Cox’s proportional hazards regression model based on the nomogram method by Ju et al.^38^ for the clinical data only.
- We evaluate the performance of the PreSANet on a holdout test set using an internal retrospective dataset acquired from a separate institution. As part of this evaluation, we test a data augmentation strategy which takes into account the source institution when splitting the data for both unseen data and domains.

## Results

### Patient cohorts

The TCIA “Head–Neck-PET–CT”^28^ dataset—referred to on-wards as TCIA—is a public dataset that contains 3D PET and CT radiotherapy planning imaging data along with clinical information and contoured regions of interest of 298 patients. Patients with histologically proven H&N cancer, receiving radiotherapy or chemoradiotherapy with curative intent between 2006 and 2014 were included. Patients with metastasis or palliative intent treatment at the outset of the study were excluded. All patients underwent a pretreatment 18F-fluorodeoxyglucose (FDG) PET/CT scan between 6 and 66 days before treatment (median: 18 days). PET scans were reconstructed using the ordered subset expectation maximization (OSEM) iterative algorithm, with photon absorption and scattering attenuation correction performed. Patient sex, age, primary tumor site, tumor classification stage, therapy choice (radiation or chemo-radiation), and presence of prior surgery were included in the clinical data. Table 1 presents the patient cohort characteristics and demographic data used as input to the model. Median follow-up from radiotherapy was 45 months (range 6–112) An imbalanced outcome distribution is clearly observable: by the end of the study, 13% of patients reported distant metastasis (median: 15 months, range 4–51), 14% presented locoregional tumor recurrence (median: 17 months, range 4–59) and 19% were reported deceased from any cause (median: 28 months, range 6–82).

**Table 1:** Characteristics of patient cohorts with H&N cancer for 4 institutions acquired from the Cancer Imaging Archive (TCIA) and internally at the CHUM.

	Internal	TCIA			
	CHUM (n = 371)	CHUM (n = 65)	CHUS (n = 101)	HGJ (n = 91)	HMR (n = 41)
**Age at diagnosis (years)**					
Mean (SD)	62 (8)	63 (9)	63 (10)	61 (11)	66 (9)
Minimum-maximum	40–85	44–90	34–88	18–84	49–85
**Sex**					
Male	289 (78%)	49 (75%)	73 (72%)	74 (81%)	31 (76%)
Female	82 (22%)	16 (25%)	28 (28%)	17 (19%)	10 (24%)
**Follow-up (months)**					
Mean (SD)	51 (23)	40 (11)	45 (18)	53 (24)	39 (20)
Minimum–maximum	1–104	12–66	18–93	25–112	6–70
DM	28 (7%)	2 (5%)	10 (10%)	14 (15%)	11 (27%)
LR	36 (9%)	7 (11%)	15 (15%)	12 (13%)	9 (22%)
OS	64 (17%)	5 (8%)	18 (18%)	14 (15%)	19 (46%)
**T-stage**					
N/A	0 (0%)	5 (8%)	0 (0%)	4 (4%)	1 (2%)
T1	82 (22%)	8 (12%)	9 (9%)	20 (22%)	2 (5%)
T2	129 (34%)	28 (43%)	44 (44%)	20 (22%)	17 (41%)
T3	95 (25%)	19 (29%)	31 (31%)	35 (38%)	12 (29%)
T4	65 (17%)	5 (8%)	17 (17%)	12 (13%)	9 (22%)
**N-Stage**					
N/A	31 (8%)	4 (6%)	38 (38%)	12 (13%)	5 (12%)
N1	37 (9%)	8 (12%)	10 (10%)	18 (20%)	4 (10%)
N2	266 (71%)	45 (69%)	50 (50%)	58 (64%)	27 (66%)
N3	37 (9%)	8 (12%)	3 (3%)	3 (3%)	5 (12%)
**M-Stage**					
N/A	0 (0%)	0 (0%)	0 (0%)	4 (4%)	0 (0%)
M0	371 (100%)	65 (100%)	101 (100%)	87 (96%)	41 (100%)
**TNM-Stage**					
N/A	0 (0%)	2 (3%)	0 (0%)	0 (0%)	0 (0%)
Stage I	5 (1%)	0 (0%)	3 (3%)	1 (1%)	0 (0%)
Stage II	12 (3%)	2 (3%)	17 (17%)	5 (5%)	3 (7%)
Stage III	41 (11%)	7 (11%)	21 (21%)	28 (31%)	5 (12%)
Stage IV	313 (84%)	54 (83%)	60 (59%)	57 (63%)	33 (80%)
**P16 Status**					
N/A	29 (7%)	41 (63%)	63 (62%)	31 (34%)	39 (95%)
+	251 (67%)	21 (32%)	25 (25%)	30 (33%)	2 (5%)
−	91 (24%)	3 (5%)	13 (13%)	30 (33%)	0 (0%)
**Tumor site**					
Oropharynx	369 (99%)	57 (88%)	72 (71%)	55 (60%)	19 (46%)
Nasopharynx	0 (0%)	2 (3%)	6 (6%)	14 (15%)	6 (15%)
Hypopharynx	0 (0%)	1 (2%)	1 (1%)	4 (4%)	7 (17%)
Larynx	0 (0%)	0 (0%)	22 (22%)	14 (15%)	9 (22%)
Other	2 (1%)	5 (8%)	0 (0%)	0 (0%)	0 (0%)

Figures are mean (standard deviation) where indicated, minimum–maximum value where indicated, and number (% of non-missing values) otherwise. The TNM staging system indicates the size and extent of the primary tumor, the number of nearby cancerous lymph nodes and whether the cancer has metastasized at the onset of the study, respectively.

A separate retrospective dataset—referred to from this point on as Internal CHUM—was retrospectively collected from the radiation oncology department of the CHUM hospital. It presents 371 patients from the same institution as one of the subsets of the “Head–Neck-PET–CT” with no overlap, treated for H&N cancer undergoing radiotherapy or chemoradiotherapy acquired between 2011 and 2019, with no overlap with the TCIA dataset. Details on this second cohort are also shown in Table 1 The same exclusion criteria as those from the “Head–Neck-PET–CT” study were applied^28^. Images acquired included 120kV CT and expert segmentations of primary tumors and organs at risk. PET data was also acquired separately for all patients as long as the acquisition was within 1 month of the CT acquisition date. PET images were reconstructed using the 3D row action maximum likelihood algorithm (RAMLA), corrected for photon absorption, scattering attenuation and rigidly registered to the matching CT. The clinical data was acquired from the radiation oncology department at the CHUM. The endpoints follow-up was performed between 1 and 104 months after the beginning of treatment (median: 51). The distribution of binary outcome events occurrence is as follows: at last follow-up, 7% of patients reported distant metastasis (mean: 18 months, range 3–71), 9% presented locoregional tumor recurrence (mean: 12 months, range 3–44) and 17% were reported deceased (median: 28 months, range 1–84).

### Ablation study

**Figure 1:**
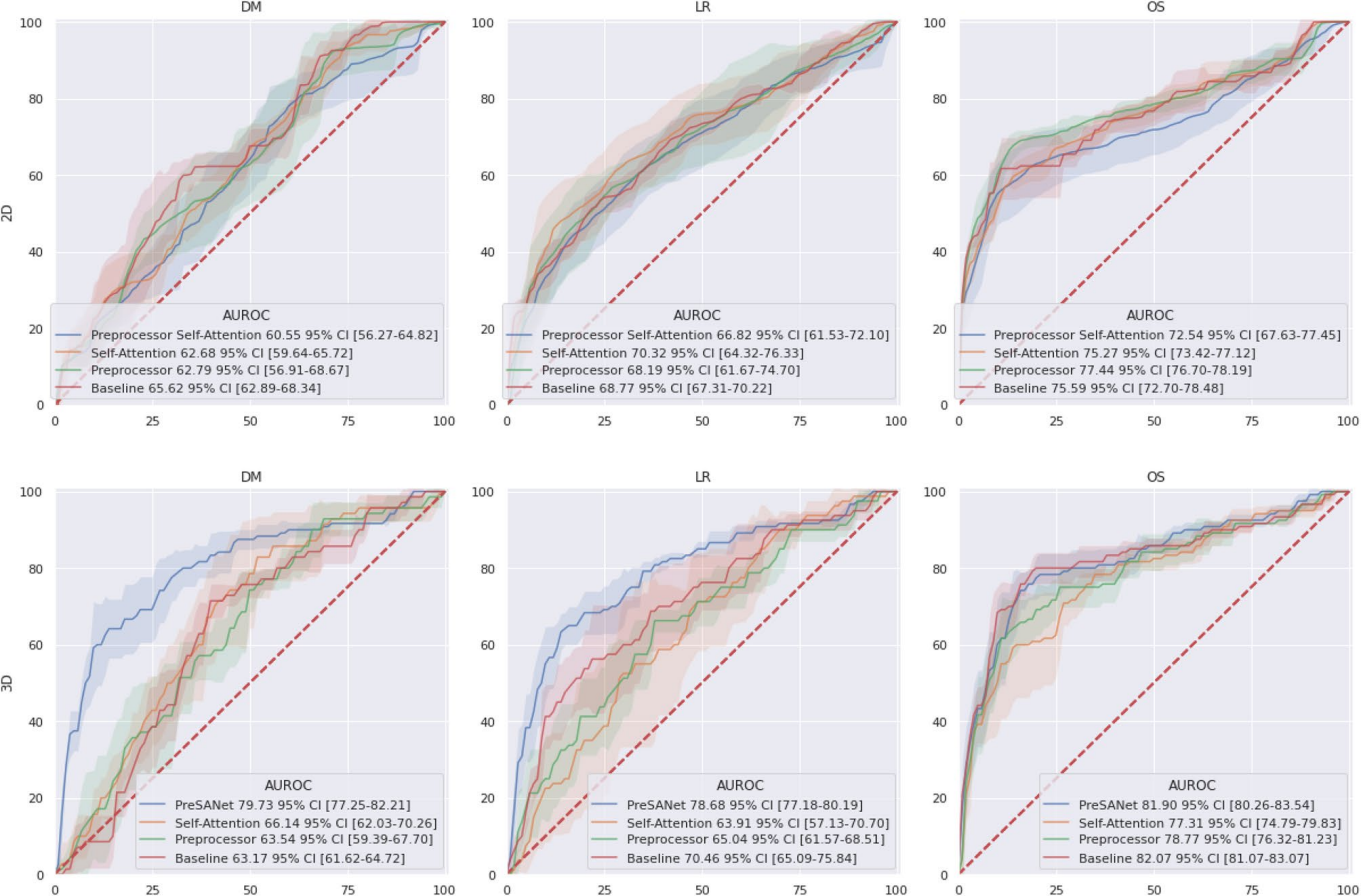
ROC curves for the proposed PreSANet model with different feature ablation. Rows represent the baseline 2D and the proposed pseudo-3D architectures. Figures presented are mean AUC [$95\%$ CI] over 5 trials. Training and validation split used is shown in Fig. 8A^30^. Figure created with seaborn/matplotlib v0.11.2 (seaborn.pydata.org).

Figure 1 summarizes the model architecture ablation results—the features tested are the pseudo-volumetric models, preprocessor module and self-attention—when using all available input modalities: CT, PET, and other clinical data. The proposed PreSANet model with all features and all input modalities shows the highest prediction performance on the test set for the three presented metrics, with an area under the receiving operating curve (AUROC) of $79.8\% [77.0-82.7]$ for DM. The performance was significantly worse when removing any combination of all three proposed features at $p < 0.05$. For LR, the model obtained an AUROC of $78.8\%$ [77.0–80.5]. Statistically significant difference between the full-featured model and the other ablation variants was found at $p < 0.05$ when removing both the preprocessor module and self-attention layers for the pseudo-volumetric variant ($70.4\%$ AUROC [64.3–76.5]), and when removing the preprocessor module for the 2D variant ($70.3\%$ AUROC [63.5–77.2]). All other variants for LR was found to perform worst than the proposed model at $p < 0.05$. For OS, an AUROC of $82.0\%$ [80.2–83.9] and an accuracy of $78.7\%$ [78.4–79.0] were obtained with no statistically significant differences found when compared with the pseudo-volumetric model without self-attention layers (AUROC = $78.8\%$ [76.0–81.6]; accuracy = $76.0\%$ [72.0–79.9]) and when compared with the pseudo-volumetric model without both the preprocessor module and the self-attention layers (AUROC = $82.15\%$ [81.0–83.3]; accuracy = $79.8\%$ [76.6–83.0]). The pseudo-volumetric variant without the preprocessor module performed worse at OS prediction at $p < 0.05$ ($77.4\%$ AUROC [74.5–80.2]). All other feature combinations showed worse performance than the proposed model for OS at $p < 0.05$. Overall, the combination of the pseudo-volumetric architecture, the preprocessor module and the self-attention layers is necessary to obtain the highest performance for the tasks of DM and LR prediction, with reduced performance observed when only a subset of features are included instead. For the task of OS prediction, the pseudo-volumetric architecture leads to significantly higher performance, with no clear improvements when including the preprocessor module and self-attention layers.

**Figure 2:**
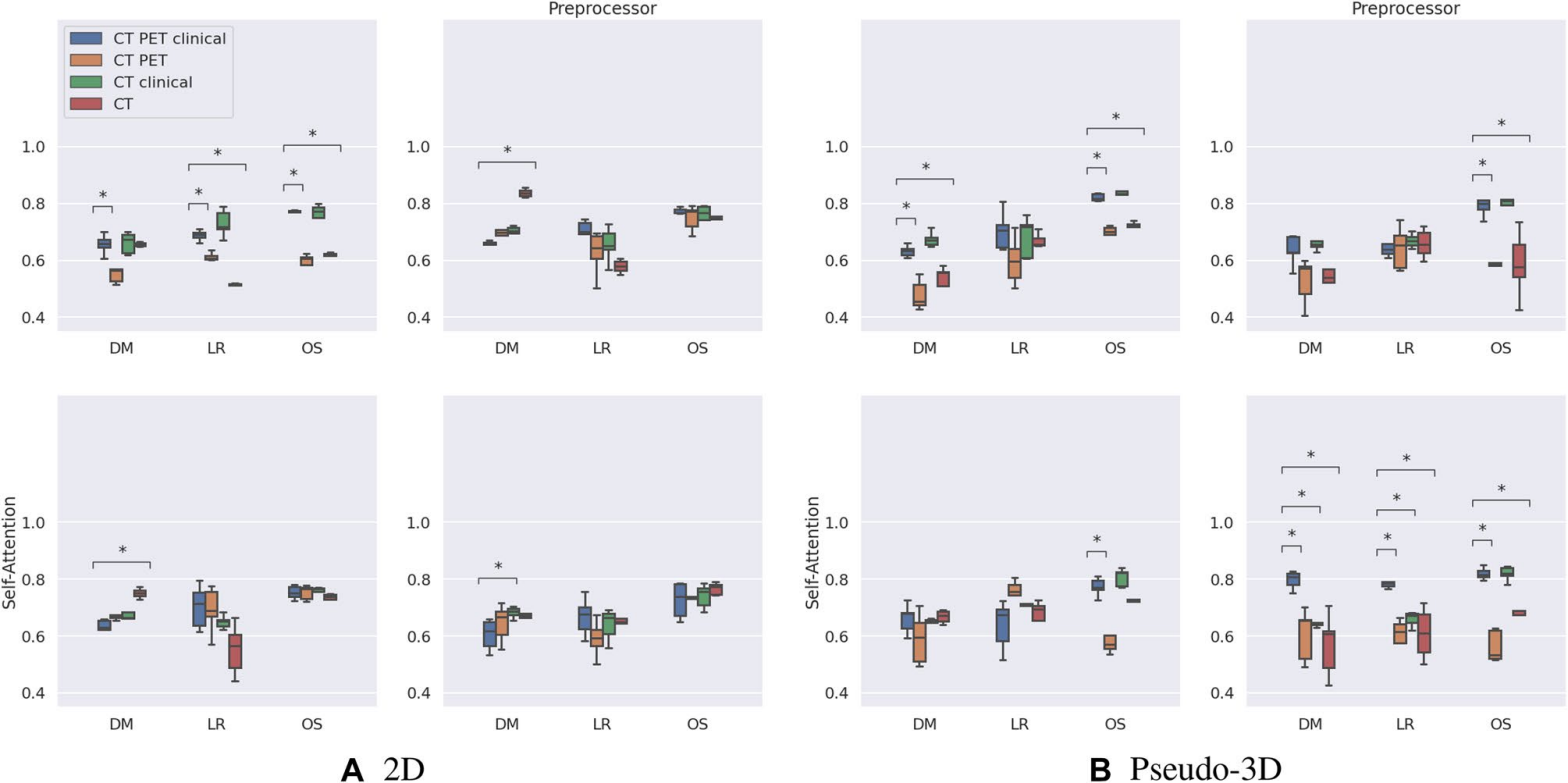
Ablation AUROC results for the proposed PreSANet model. Subfigure grids represent ablations of the Preprocessor and self-attention features for the (**A**) 2D variant, and (**B**) pseudo-volumetric variant. Within each sub-figure, the combination of input imaging and clinical modalities are presented. Figures presented are mean performance over 5 repeated trials with error bars representing $95\%$ CI. Stars indicate statistical significance using the dependent t-test for paired samples with Bonferroni correction, with the number of stars indicating: (1) $p < 0.05$, (2) $p < 0.01$, (3) $p < 0.001$, (4) $p < 0.0001$. Each modality combination is compared with the proposed model that uses CT image, PET image and clinical data as inputs. Training and validation split employed matched the one presented by Vallieres et al.^30^. Figure created with seaborn/matplotlib v0.11.2 (seaborn.pydata.org)

Figure 2 presents ablation results for the possible input modalities (PET and clinical data). In every case, the CT is included as a base input image. More specifically, a sub ablation experiment was run on the following possible model inputs: CT, CT + PET, CT + clinical data, and CT + PET + clinical data (the proposed approach). For the proposed pseudo-volumetric PreSANet model, using three input modalities shows the highest AUROC performance when predicting DM ($79.8\%$ [77.0–82.7]) and LR ($78.8\%$ [77.0–80.5]) at $p < 0.05$. For OS of the same proposed model, an AUROC of $82.0\%$ [80.2–83.9] was shown to not be a statistically significant improvement for the variant including CT and clinical data only as inputs ($82.0\%$ [79.7–84.3]). Otherwise, all other input combinations were shown to perform significantly worse at $p < 0.05$. More ablation results are presented in Table S2 in the appendix.

For the pseudo-volumetric model with self-attention, the only loss in performance due to input modality ablation was observed for OS when either removing the clinical data ($82.0\%$ [80.2–83.9] > 56.5% [51.5–61.5] AUROC). Similar behavior can be observed for the pseudo-volumetric model with the preprocessor module by either removing the clinical data when predicting OS ($78.8\%$ [76.0–81.6] > 59.5% [52.4–66.6] AUROC) or using only CT as input features ($78.8\%$ [76.0–81.6] > 58.7% [48.2–69.1] AUROC). This is also true for the baseline pseudo-volumetric model when either removing the clinical data for DM ($63.2\%$ [61.5–65.0] > 47.9% [43.2–52.6] AUROC) or OS ($63.2\%$ [61.5–65.0] > 70.3% [69.0–71.6] AUROC) or using only CT as input features for DM ($82.2\%$ [81.0–83.3] > 54.3% [51.5–57.2] AUROC) or OS ($82.2\%$ [81.0–83.3] > 71.8% [69.7–73.9] AUROC). Overall, the proposed model with both the preprocessor module and self-attention presents the best performance when pairing the CT input with both the PET and the clinical data. Table S3 (in appendix) presents the ablation performance of the pseudo-volumetric model on the holdout HMR and CHUM datasets.

For the 2D PreSANet model, a statistically significant performance gain can be observed for predicting DM when removing PET from the inputs ($60.6\%$ [55.7–65.5] < 68.3% [66.6–70.1] AUROC). All other input combinations presented equivalent performance with $p \ge 0.05$. For the 2D model with self-attention, a similar performance gain was observed for DM when removing additional PET and clinical data, where performance improved with the removal of features instead of a decrease. For LR and OS, no statistically significant difference was found with the modalities ablation. For the 2D model with the preprocessor module, a significant gain in model performance was observed for the DM prediction task when removing both PET and clinical data from the inputs ($62.8\%$ [56.1–69.5] < 83.8% [82.5–85.0]). For LR and OS, no statistically significant difference was found with the modalities ablation. For the baseline 2D model, statistical significant evidence of performance loss due to the removal of the clinical data from the inputs can be observed for DM trained on CT and PET ($65.7\%$ [62.6–68.8] > 54.9 [52.5–57.2]), for LR trained on CT and PET ($68.8\%$ [67.1–70.4] > 61.3% [60.0–62.5]) or trained on just CT ($68.8\%$ [67.1–70.4] > 51.6% [51.3–51.8]), and for OS trained on CT and PET ($75.7\%$ [72.4–79.0] > 59.3% [56.3–62.3]) or trained on just CT ($75.7\%$ [72.4–79.0] > 62.1% [61.6–62.6]). The baseline 2D model with CT as its only input is equivalent to the approach by Diamant et al.^31^. Table S4 (in appendix) presents the ablation performance of the 2D model on the holdout HMR and CHUM datasets. Overall, the 2D model variants seem to be confounded with the addition of PET or clinical data.

### Adding PET to the prediction model

**Figure 3:**
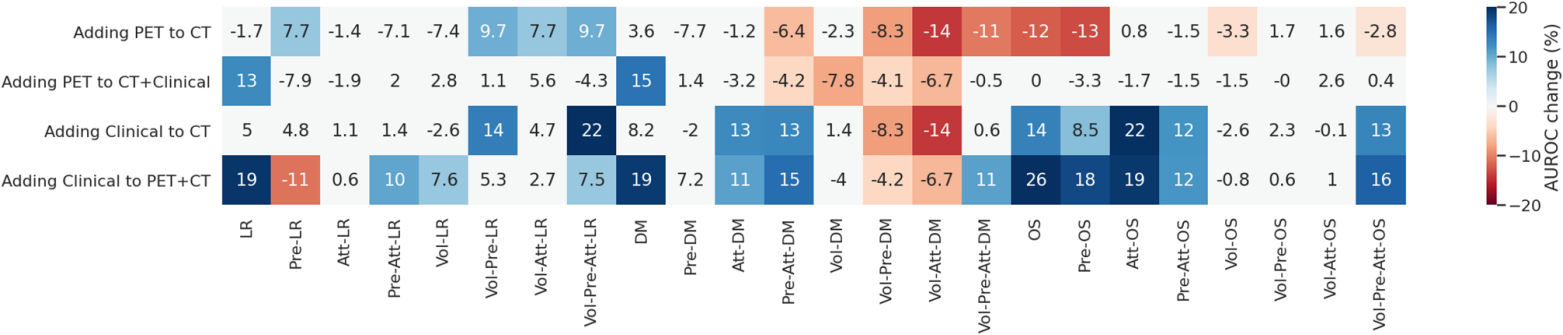
Effect of adding clinical data or PET images combined to the baseline CT. Columns represent different architectures used in the ablation experiments, for each of the predicted targets. Figures are percentage change in mean AUROC with the addition of the indicated modality to the indicated base modalities. Figures and colors represent the effect size for cases where a statistical significance was found (*p* value < 0.05). Squares in white indicate no statistically significant difference was found. Figure created with seaborn/matplotlib v0.11.2 (seaborn.pydata.org)

Figure 3 shows the effects of the addition of either modalities individually on model performance across all ablation experiments. With the addition of PET, out of 48 total experiments, 12 cases showed a statistically significantly worse performance at $p < 0.05$ (mean: $7.8\%$ AUROC, range 3.3–14.3), and a statistical improvement in 6 cases (mean: $10.5\%$ AUROC, range 7.7–14.7). For clinical data, an improvement was observed in 22 cases (mean: $14.7\%$ AUROC, range 7.5–25.6); a loss in performance was observed in 5 cases (mean: $8.8\%$ AUROC, range 4.2–14.2). No statistical significance was found in all other experiments.

Due to the instability resulting from the introduction of PET data as shown in the ablation studies, an experiment was conducted to investigate the viability of this modality in the Internal CHUM dataset. The performance of the model trained using a randomized 5-fold cross-validation on this dataset, comparing the effects of adding PET to the inputs showed a marked decrease in performance across all relevant metrics (see Table S1 in Appendix). With the addition of PET to CT + clinical inputs, a 3.7–11.8% decrease in prediction accuracy and 0.6–10.5% decrease in AUROC across all three outcomes. For this reason, all of the subsequent results use CT + clinical as input modalities.

### Internal and external validation

**Figure 4:**
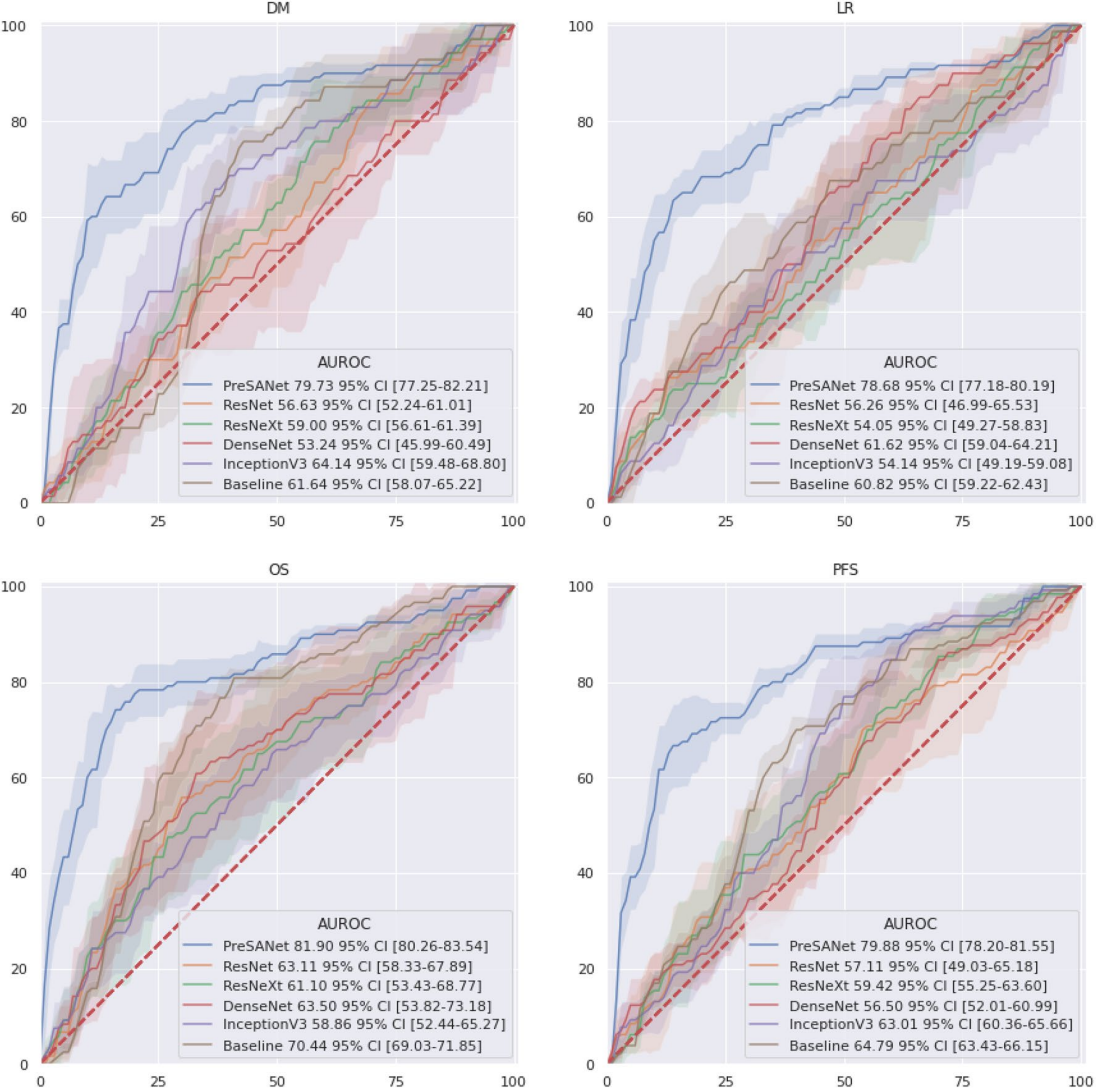
ROC curves for the proposed PreSANet model compared with previous works and off-the-shelf models. Figures presented are mean AUC [$95\%$ CI] over 5 trials. Training and validation split used is shown in Fig. 8A^30^. Figure created with seaborn/matplotlib v0.11.2 (seaborn.pydata.org)

Figure 4 shows the AUROC internal validation classification performance (see Fig. 8A), which corresponds to previous deep learning approach^31^ and radiomics approach^30^ by PreSANet as well as popular state-of-the-art off-the-shelf models. As a baseline, a simple CNN as described by Diamant et al.^31^ was also trained and evaluated. Overall, the proposed method achieved the highest AUROC for all three outcomes (DM: $79.83\%$ [77.05–82.60]; LR: $78.8\%$ [77.1–80.4]; OS: $82.0\%$ [80.2–83.9]) compared to the baseline as well as InceptionV3, DenseNet, ResNet and ResNeXt. The shaded confidence interval shows that most of these approaches perform similarly statistically whereas the proposed approach performs significantly better. Furthermore, the prediction result of the progression-free survival (PFS) outcome, combining both DM and LR into a single label is shown, using the combination of the prediction probabilities of each individual DM and LR models. The proposed PreSANet model achieved $79.9\%$ AUROC [78.2–81.6], surpassing all other compared models, as well as surpassing both DM and LR models separately.

Table 2 describes the proposed model evaluated on split A, compared with the previous works by Vallières et al.^30^ using a random forest method and Diamant et al.^31^ using a CNN approach. As well, popular state-of-the-art off-the-shelf models were also trained and evaluated on the same data split are described. Finally, a baseline CNN model was trained using the architecture and hyperparameters described in^31^.

**Table 2:** Model comparison with previous works.

Model	Metric	DM	LR	OS
Cox Model^38^	Accuracy	58.0 [58.0–58.0]	53.1 [53.1–53.1]	73.8 [73.8–73.8]
	Specificity	**94.6 [94.6–94.6]**	**100.0 [100.0–100.0]**	76.8 [76.8–76.8]
	Sensitivity	21.4 [21.4–21.4]	6.2 [6.2–6.2]	70.8 [70.8–70.8]
	AUROC	71.8 [71.8–71.8]	70.8 [70.8–70.8]	72.0 [72.0–72.0]
Random forest^30^	Accuracy	**77.4 [68.6–81.6]**	67.5 [61.3–73.8]	65.6 [59.7–69.2]
	Specificity	76.0 [58.4–82.2]	68.4 [60.9–80.1]	57.6 [50.2–67.4]
	Sensitivity	**86.6 [76.2–91.4]**	63.7 [57.4–73.5]	76.2 [70.3–87.9]
	AUROC	**86.5 [77.4–94.5]**	69.8 [58.1–79.3]	78.3 [64.5–88.0]
CNN^31^	Accuracy	63.4 [59.3–67.6]	62.3 [60.0–64.6]	70.3 [68.2–72.3]
	Specificity	62.6 [58.9–66.3]	74.7 [57.3–92.0]	63.9 [56.6-71.2]
	Sensitivity	64.3 [53.4–75.1]	50.0 [31.4–68.6]	76.7 [71.8–81.6]
	AUROC	61.6 [57.6–65.6]	60.8 [59.0–62.6]	70.5 [68.9–72.1]
DenseNet	Accuracy	51.3 [42.7–60.0]	57.5 [55.0–60.1]	59.6 [49.4–69.9]
	Specificity	67.0 [36.8–97.1]	37.6 [24.7–50.5]	73.4 [61.8–85.0]
	Sensitivity	35.7 [10.7–60.8]	37.5 [24.7–50.5]	45.8 [20.3–71.4]
	AUROC	53.1 [45.1–61.3]	61.7 [58.8–64.5]	63.5 [52.7–74.3]
InceptionV3	Accuracy	58.7 [54.8–62.5]	53.9 [52.8–55.0]	56.4 [51.2–61.7]
	Specificity	55.9 [32.0–78.8]	59.1 [45.3–73.0]	75.4 [66.4–84.3]
	Sensitivity	61.4 [37.8–85.1]	48.8 [35.1–62.4]	37.5 [19.8–55.2]
	AUROC	64.2 [58.9–69.4]	54.1 [48.5–59.6]	58.9 [51.7–66.0]
ResNet	Accuracy	52.5 [48.2–56.8]	55.5 [49.3–61.7]	59.0 [53.9–64.1]
	Specificity	50.7 [22.8–78.5]	52.2 [31.5–72.9]	58.8 [35.2–82.4]
	Sensitivity	54.9 [24.7–83.9]	58.8 [41.6–75.9]	59.2 [36.5–81.9]
	AUROC	56.7 [51.8–61.6]	56.3 [45.9–66.6]	63.1 [57.8–68.4]
ResNeXt	Accuracy	56.9 [51.2–62.7]	54.5 [52.6–56.3]	56.8 [50.8–62.8]
	Specificity	32.4 [16.3–48.4]	62.7 [43.8–81.5]	67.8 [47.8–87.8]
	Sensitivity	81.4 [68.4–94.4]	46.2 [27.8–64.7]	45.8 [22.9–68.8]
	AUROC	59.0 [56.3–61.6]	54.0 [48.7–59.3]	61.1 [52.5–69.7]
PreSANet	Accuracy	74.5 [70.9–78.1]	**74.2 [71.6–76.8]**	**78.7 [78.4–79.0]**
	Specificity	84.8 [80.2–89.6]	81.0 [72.5–89.5]	**80.7 [73.4–88.1]**
	Sensitivity	64.2 [56.6–71.7]	**67.5 [61.5–73.5]**	**76.7 [69.6–83.8]**
	AUROC	79.8 [77.1–82.6]	**78.8 [77.1–80.4]**	**82.0 [80.2–83.9]**

Models were evaluated on split A, corresponding to the training and evaluation split presented by Vallieres et al.^30^. Off-the-shelf models were trained on a 2D image of the largest lesion slice, computed the same way as in the ablation study. Nomogram model^38^ was calculated on the clinical data only. To obtain a 3 channel image required by these models, two repeated CT images and the PET image were concatenated. Figures reported are mean [95% Confidence Interval] for each of the 5 evaluated metrics, where available. Accuracy metric is normalized per class frequency.

Bold corresponds to the highest value across Models for each Metric (Accuracy, Specificity, Sensitivity, AUROC) for each outcome (DM, LR, OS).

The proposed PreSANet approach shows the highest accuracy and AUROC for both LR (accuracy: $74.2\%$ [71.6–76.8]; AUROC: $78.8\%$ [77.1–80.4]) and OS (accuracy: $78.7\%$ [78.4–79.0]; AUROC: $82.0\%$ [80.2–83.9]). It also shows high imbalanced classification performance for OS (specificity: $81.0\%$ [72.5–89.5]; sensitivity: $67.5\%$ [61.5–73.5]). For LR, a higher specificity was achieved by the traditional Cox’s proportional hazard approach^38^ at $100\% > 81.0\%$ [72.5–89.5], but with very poor sensitivity $6.2\% < 67.5\%$ [61.5–73.5]. A higher overall sensitivity was achieved by the CNN^31^ at $77.5\%$ [69.2–85.8] > 67.5% [61.5–73.5], trading off however for a lower specificity at $37.6\%$ [24.7–50.5] < 81.0% [72.5–89.5].

For DM, the highest performing model remains the Random Forest approach by Vallières et al.^30^, which compared to our approach obtained a balanced accuracy of $77.5\%$ [68.6–81.6] > 74.5% [70.9–78.1], an AUROC of $86.5\%$ [77.4–94.5] > 79.8% [77.1–82.6] and a sensitivity of $86.6\%$ [76.2–91.4] > 64.2% [56.6–71.7]. The highest specificity for DM was achieved by the traditional Cox’s model^38^ with $94.6\% > 84.8\%$ [80.2–89.6].

Table 3 presents the performance of our proposed PreSANet approach on an external dataset (see Fig. 8B for data split description), when trained entirely on the TCIA dataset. The AUROC achieved for DM at $68.8\%$ [68.0–69.7], for LR at $69.2\%$ [68.1–70.3] and for OS at $69.3\%$ [68.4–70.1] shows a very small $95\%$ confidence interval across 5 repeated trials.

**Table 3:** Performance on the Internal CHUM dataset.

Test set	Metric	DM	LR	OS
Internal CHUM	Accuracy	63.9 [63.4–64.4]	64.5 [63.7–65.3]	64.0 [63.6–64.4]
	Specificity	75.3 [70.6–80.0]	70.2 [60.2–80.3]	73.0 [57.6–88.3]
	Sensitivity	52.5 [47.8–57.2]	58.8 [48.2–69.4]	55.0 [40.2-69.8]
	AUROC	68.8 [67.9–69.7]	69.2 [68.0–70.4]	69.3 [68.3–70.2]

Training is performed in the entirety of the TCIA dataset, with 298 samples. Testing is performed on the Internal CHUM dataset, with 371 samples. In both training and testing sets, the CHUM institution was seen by the model. Accuracy is normalized per class frequency.

### Testing cross-site generalization

**Figure 5:**
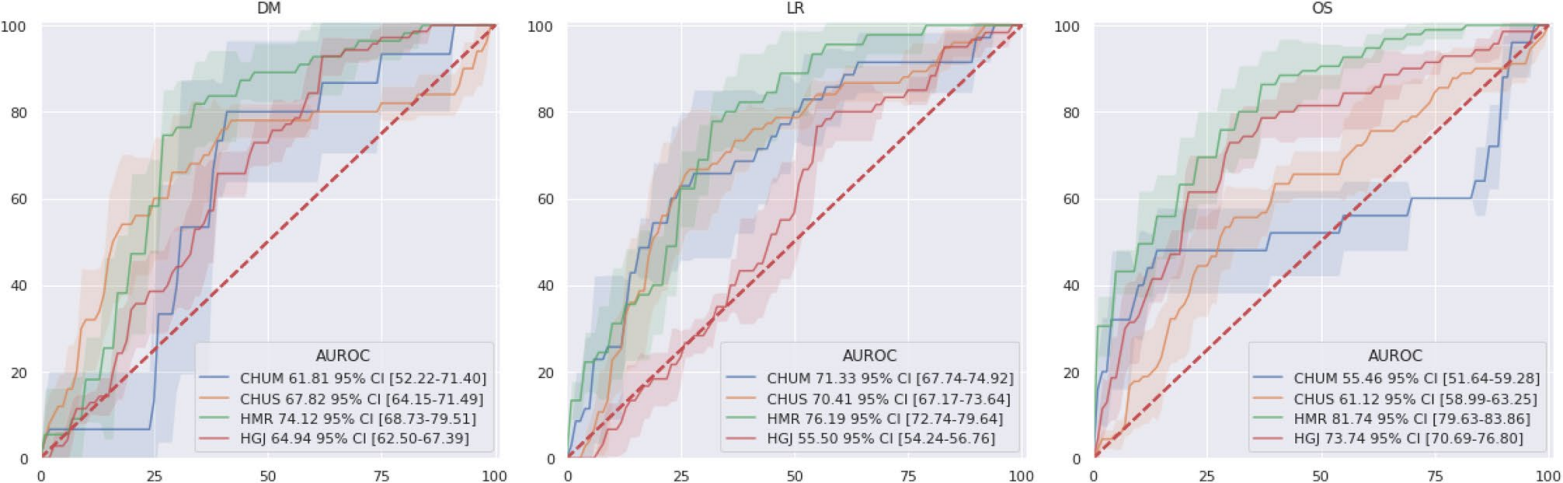
ROC curves for the proposed PreSANet evaluated in a single-site cross-validation. Figures presented are mean AUC [$95\%$ CI] over 5 trials. Training and validation used is shown in Fig. 8C. Figure created with seaborn/matplotlib v0.11.2 (seaborn.pydata.org).

Figure 5 presents the 4-fold cross-validation ROC curves for each of the evaluated sites. Each of the folds contained three sites in training and one hold-out site for testing. Across all possible outputs, the highest performing test fold was that of HMR with $74.1\%$ [68.7–79.5] for DM, $76.2\%$ [72.7–79.6] for LR and $81.7 \%$ [79.6–83.9] for OS. Spearman’s correlation factor between training set size and AUROC performance was found to be 0.753 for DM, $-\,0.150$ for LR and 0.772 for OS. The worst performing folds with little overlap in the 95% confidence interval curves were HGJ for LR (AUROC: $55.5\%$ [54.2–56.8]) and CHUM for OS (AUROC: $55.5\%$ [51.6–59.3]).

Table 4 presents the cross-validation performance with the addition of balanced accuracy, specificity and sensitivity metrics. For DM, the highest sensitivity is obtained when testing on CHUM at $86.7\%$ [70.7–100.0] at the cost of low specificity at $57.4\%$ [45.7–69.1] < 68.1% [60.6–75.7 obtained for CHUS. For LR, a sensitivity of $88.9\%$ [79.2–98.6] was obtained for HMR, yet resulted in a specificity of $56.3\%$ [46.0–66.5] < 73.0% [61.1–85.0] when compared with CHUS, which had the highest specificity. The fold tested on HMR for OS achieves the highest balance of specificity ($70.9\%$ [62.2–79.6]) and sensitivity ($86.3\%$ [78.6–94.0]) as well as the highest sensitivity. The mean AUROC cross-validation performance was $67.2\%$ [61.3–73.1] for DM, $68.4\%$ [65.1–71.6] for LR and $68.1\%$ [64.9–71.2] for OS. The mean balanced accuracy obtained was 71.6% [68.1–75.2], $69.6\%$ [66.2–73.0] and $71.2\%$ [69.3–73.1] for DM, LR and OS.

**Table 4:** Per-site 5-fold cross-validation. Figures reported are mean [95% confidence interval] for each of the 5 evaluated metrics.

Test set	Metric	DM	LR	OS
CHUS	Accuracy	73.1 [70.5–75.6]	7.18 [68.0–74.3]	65.5 [64.9–66.0]
	Specificity	**68.1 [60.6–75.7]**	**73.0 [61.1–85.0]**	63.1 [49.09–77.2]
	Sensitivity	78.0 [74.1–81.9]	69.3 [62.7–76.0]	67.8 [53.4–82.1]
	AUROC	67.85 [63.7–72.0]	70.4 [66.8–74.0]	61.2 [58.8–63.5]
CHUM	Accuracy	72.0 [68.0–76.1]	**72.8 [67.8–77.9]**	67.5 [64.3–70.7]
	Sensitivity	**86.6 [70.7–100.0]**	74.3 [58.0–90.6]	48.0 [38.4–57.6]
	Specificity	57.4 [45.7–69.1]	71.4 [59.5–83.3]	**87.0 [80.4–93.7]**
	AUROC	61.9 [51.2–72.6]	71.3 [67.3–75.3]	55.4 [51.1–59.7]
HGJ	Accuracy	66.2 [63.7–68.6]	62.0 [59.7–64.2]	73.1 [71.2–75.0]
	Sensitivity	78.6 [58.8–98.4]	78.3 [71.8–84.9]	77.1 [71.9–82.4]
	Specificity	53.8 [38.6–69.0]	45.6 [37.7–53.5]	69.1 [63.7–74.5]
	AUROC	64.9 [62.2–67.7]	55.5 [54.1–56.9]	73.8 [70.3–77.2]
HMR	Accuracy	**75.2 [70.0–80.3]**	72.6 [69.4–75.7]	**78.6 [76.7–80.5]**
	Sensitivity	83.6 [73.3–94.0]	**88.9 [79.2–98.6]**	**86.3 [78.6–94.0]**
	Specificity	66.7 [62.1–71.3]	56.3 [46.0–66.5]	70.9 [62.2–79.6]
	AUROC	**74.0 [68.0–80.0]**	**76.3 [72.4–80.1]**	**81.9 [79.6–84.3]**
**Average**	Accuracy	71.6 [68.1–75.2]	69.6 [66.2–73.0]	71.2 [69.3–73.1]
	Sensitivity	81.7 [69.2–94.3]	77.7 [67.9–87.5]	69.8 [60.6–79.0]
	Specificity	61.5 [51.7–71.3]	61.6 [51.1–72.1]	72.5 [63.8–81.2]
	AUROC	67.2 [61.3–73.1]	68.4 [65.1–71.6]	68.1 [64.9–71.2]

Each test set indicates that samples belonging to it were used for testing: the rest of the data was used for training and validation. Accuracy metric is normalized per class frequency.

Bold corresponds to the highest value across Models for each Metric (Accuracy, Specificity, Sensitivity, AUROC) for each outcome (DM, LR, OS).

## Discussion

Reliable forecasting of radiotherapy outcomes remains a challenging task for clinicians. Accurate predictions could not only help in current clinical practice but it could also help physicians tailor treatment plans to individual anatomies and physiologies. Improved prediction technologies could help guide the physician’s decision on treatment intensification and omitting systemic for local therapy plans, all of which would help limit side effects from treatment, improve quality of life, improve treatment efficiency in terms of the delivered dose, and reduce radiation exposure and cost to the patient. In this study, a pseudo-volumetric CNN-based deep learning model is proposed that combines PET, CT, and patient clinical data to predict DM, LR, and OS for head and neck cancer cases undergoing radiotherapy.

While our work is not the first to have explored using CNNs for tumor risk assessment^31^, the proposed model introduces three main novel features: a preprocessor module that normalizes image differences across institutions, self-attention layers to identify salient latent space image information, and a pseudo-volumetric formulation that allows the model to use all slices from a scan volume even when the number of slices is variable^33^. Indeed, a major limitation of deep learning approaches in medical imaging is the lack of sufficient sample points necessary for neural networks to generalize. Compared with previous approaches, our proposed architectural features attempt to offset this issue by extracting more information from the available data: a beneficial alternative when acquiring more data becomes too difficult. The self-attention and preprocessor module blocks are recent techniques employed in state-of-the-art deep learning approaches for natural and medical image processing, respectively. Despite their black box nature, both additions showed improved training performance without increasing model capacity—929,678 trainable parameters in the baseline 2D CNN from the ablation study and the internal validation, and 881,014 trainable parameters in PreSANet—indicating that the architectural constraint helped guide the training process itself instead of increasing the memorization capabilities of the model. Furthermore, ablation experiments suggest an interaction effect between both features, possibly from the learned saliency maps requiring a specific normalized image.

Introducing another imaging dimension—along the anatomical vertical axis in our case—adds exponential complexity to DNNs, quickly becoming intractable computationally. Furthermore, as is the case of patient scans, the depth dimension is often of variable length, adding another source of difficulty for rectangular arrays requiring neural networks. A popular technique to address this is to construct a fully connected two dimensional CNN and take the average or maximum prediction score across all slices. A more complex approach lies in using these prediction scores as inputs to a secondary classifier network, allowing a learned pooling method, as was used by the AI system in McKinney’s UK and US clinical trials for screening mammography to great success^39^. Our proposed pseudo-volumetric can thus be seen as a natural extension of these approaches, integrating the secondary network in an end-to-end fashion directly on the latent representation instead of prediction classes. This, along with the use of both mean and variance as statistics, allows robustness to inter-patient anatomical variability, but also potentially to missing slices within a patient. Nonetheless, our approach is likely overly cautious, processing only slices containing the primary tumor. Exploring the impact of whole body scans could lead to improved performance if analysis of distant anatomical structures is made available to the model; this is especially true for metastasis prediction.

One benefit of medical imaging over natural images is the ability to obtain complementary scans of the same patient and region of interest. Thus, we explored the impact of adding information via PET imaging, as well as patient clinical information. To explore the impact of this complementary information, each of the previously explored ablation model variants was tested with various combinations of additional information on top of the baseline CT. It should be noted that the baseline 2D CT-input model is equivalent to the previous CNN approach^31^. Interestingly, the features proposed in the PreSANet model enabled it to make use of the additional input data whereas all other pseudo-volumetric variants were only partially sensitive to multimodal information. Adding clinical data on the other hand led to an improvement in $46\%$ of the experiments, while a loss was observed in only 5 out of 48 ablation cases. As comorbidities that affect treatment efficacy are included in this data, our model can make use of this additional information to improve overall prediction for both regional and systemic outcomes. In a quarter of the cases, the addition of PET lead to a significant improvement in predictive performance. With a baseline of CT and clinical data, an improvement was seen in only 2 cases, but with the highest percentage increase at $+\,14\%$ AUROC. Most of these seem to happen by adding PET to CT only inputs, where the extra imaging information becomes beneficial for local recurrence outcome prediction. However for distant metastases and overall survival OS prediction, the addition of PET to either CT or CT + clinical inputs lead to a significant loss in performance in a third of the ablation configurations. As both metastasis and death are both systemic outcome of treatment, information extracted from PET seems to be more beneficial in predicting local tumor behavior after radiotherapy. This concurs with the functional usage of PET to measure changes in metabolic process of tumors, a local physiological activity. While this seems to contradict at first with findings in literature for similar applications, the most important difference to note is that the imaging information provided to expert clinicians for assessment is in the form of whole-body FDG PET/CT^40–42^. With a systemic view of the post-treatment effects, physicians can better predict the progression of H&N cancers. In this particular application, the datasets were restricted to the field of view of the CT—that is of the head and neck regions—thus limiting the type of regional or distal metabolic activity information that the model could extract from PET images. Furthermore, while part of this information should be recovered in the clinical data made available to the model in the form of TNM staging, the exclusion criteria for this study specifically selects patients without onset distant metastasis (M) staging. Finally, there are the challenges associated specifically with PET as a modality. Indeed, the amount of variability in PET tracers and sequences when considering multicenter datasets^43^, as well as the poor geometric accuracy associated with this modality^44^ all make it much more difficult for learning algorithms to extract relevant information. It is likely therefore that a combination of these reasons lead to the information poor effects of PET imaging inclusion for predicting systemic outcomes such as death and metastasis. To verify if these effects were also present in the Internal CHUM dataset, and to decide if adding PET to the subsequent experiments was worthwhile, an experiment was conducted to explore the predictive value of including PET in the Internal CHUM dataset. Overall, the 5-fold cross-validation showed significantly lower performance with the inclusion of PET. Compared with the TCIA dataset where CT and PET were acquired together via the 18-FDG PET/CT acquisition scheme, ensuring precise anatomical correspondence, PET from the Internal CHUM dataset were selected as long as the acquisition time was within 1 month of the CT. While rigid registration and CT attenuation preprocessing steps were both present, many other acquisition parameters in the Internal CHUM dataset were not controlled for between patients such as the choice of radio-tracers, timing variance, method of injection, increasing the intra-dataset variability. The difficulties in obtain a uniform distribution of functional PET for inter-dataset normalization made the information less exploitable with regards to PreSANet’s empirical performance. Combined with the benefits of PET being limited to regional outcomes, our proposed model benefited more from clinical data than PET, which guided the decision of using a combination of CT and clinical data.

The choice of the data splitting strategy was made to allow comparison with other classification works on the same dataset. Indeed, our method shows a significant improvement over both the random forest^30^ and CNN methods^31^ for recurrence ($7.2\%$ accuracy and $9.8\%$ AUROC improvement) and survival prediction ($10.7\%$ accuracy and $4.0\%$ AUROC improvement). Furthermore, the 95% confidence interval for these metrics shows no overlap with the reported results from either works, indicating the importance of the improvement the pseudo-volumetric multi-modal method allows. In terms of true positive and negative predictive rate, PreSANet improved mainly over the specificity of the model ($13.0\%$ increase for LR and $13.7\%$ increase for OS), which is particularly important clinically as recurrence and death events are significantly rarer. However, PreSANet has more difficulty predicting metastasis, showing comparable performance with the random forest method with an accuracy 95%CI of [70.9–78.1] = $77\%$ and an AUROC 95%CI of [77.1–82.6] < 86%. Compared with the simple CNN^31^, a $13.5\%$ accuracy loss and $8.2\%$ AUROC loss was observed. Given that both our method and the random forest method perform its image analysis on multi-modal inputs, there seems to be a benefit to information sparsity for metastasis prediction.

The final section of this study explored the capacity of proposed PreSANet model to predict treatment outcomes across different sites. To do this, a per-site 4-fold cross-validation experiment was conducted in which each of the available sites—CHUS, HGJ, HRM, and CHUM—was held out for testing, with the remaining combined multi-site dataset used for model training. In all of the folds, the model was trained only with CT and clinical data as inputs, given the performance instability introduced by PET. It could be expected that given the uneven distribution of sample across sites (the CHUM having a combined count of 518 testing samples while the HMR site only having 41 testing samples), a larger training set would result in higher performance. While no Spearman’s correlation was found for LR ($-\,0.150$) between performance from CHUM and HMR, a noticeable one can be observed for DM (0.753) and OS (0.772). Indeed, the training dataset difference is the most extreme in this case at 233 versus 628, respectively, yielding a performance difference of $12.8\%$ AUROC and $3.2\%$ accuracy for DM, and $26.5\%$ AUROC and $11.1\%$ accuracy for OS. While this does suggest the importance of training set size, as supported by the literature across disciplines^45–47^, it appears dependent on the target outcome. Nonetheless, the best performance overall was observed in the HMR fold for both accuracy and AUROC across all three outcomes. Multi-site generalization seems possible by our approach as the performance of the folds seem to even show comparable results to the external validation case (see Table 3), where the CHUM testing site was present in both training and testing sets. Indeed the CHUM fold only shows a $2.1\%$ AUROC increase for LR prediction, despite not benefiting from previously sites. Overall, PreSANet shows a prediction power averaging $71.6\%$, $69.6\%$ and $71.2\%$ balanced accuracy for DM, LR and OS respectively. It also display strong negative predictive rate with $81.7\%$, $77.7\%$ and $69.8\%$ sensitivity at the cost of specificity, which remains difficult due to the imbalanced nature of the datasets.

One of the current limitation of this study is on the clinical impact of our predictive model. Indeed, with the goal of risk stratification, where physicians could decide on more specialized treatment techniques, e.g. local versus systemic radiation, with or without surgery or chemotherapy, overall outcome classification doesn’t yet allow this patient categorization. A variant of the model for a regression problem, predicting time to recurrence, metastasis or death of the patient would improve the clinical value of this work. Another limitation is the lack of control for the type of therapy administered in the training dataset; in this study a mix of radiotherapy and chemo-radiotherapy was used. While our current work is mainly limited by the overall number of available samples, further work either restricting the specific type of therapy, the dose administered, the choice of regional or systemic treatment and the presence of prior surgery could be interesting to explore. Alternatively, adding this information directly as inputs could improve model performance too. Finally, the question of whether adding PET leads to significant change remains a nuanced one. While there was enough cases in the ablation that showed worse performance for us to forgo including this data in the multi-dataset experiments, there remain nonetheless some configurations showing promising results. Overall, the multi-modal inputs had varied effects on the proposed architecture and should explored more.

This work demonstrates that for combined multi-site imaging datasets, certain techniques can be used to improve deep learning model performance for tumor outcome prediction tasks. In a set of ablation experiments on the public multi-site TCIA Head–Neck-PET–CT dataset, statistically significant performance improvement was demonstrated when combining all proposed features and all input modalities. In every case, training was performed using the training split used in previous literature for this particular dataset, with transfer learning employed across sites as well as using repeated 5 trials for increased statistical power. To validate the proposed approach, a multi-site dataset combining the aforementioned dataset with a novel retrospective head and neck dataset, achieving generalization across sites. The per-single site cross-validation experiments demonstrated the model’s prediction capabilities on a larger unseen validation set, with this validation scheme chosen specifically to demonstrate the cross-site generalizability potential of the model, something which isn’t quite as confidently shown in the more traditional single site hold-out test approach. Overall, we found that the results supported the primary goal of this study, which was to demonstrate the applicability of the proposed model when tested on site specific samples, after it was trained on a variety of sites. This is to better reflect the real world application where specialists from a particular institution would like to fine-tune this model on a potentially larger dataset of their own acquisition, despite its results being fine-tuned on different data sources. Future study will look at improving on the PET modality integration for improved performance, as well as performing an analysis on latent space of the pseudo-volumetric embedding since this encoding is similar to that of variational autoencoders.

## Methods

### Data preparation

Imaging data from the patient cohorts originated in DICOM format containing both PET and CT volumes of shape $512\times 512$. Slice depth distribution ranged between 83 and 348 (median: 132). Isotropic resampling was performed to obtain a uniform $1\times 1$mm spacing between pixels. To lower memory consumption during model training, the resolutions were downscaled in the axial plane to $196\times 196$. PET were standardized to obtain the standard uptake value (SUV) while CT were converted to Hounsfield scale (HU). Each modality was then normalized to obtain a pixel intensity distribution with zero mean and unit standard deviation. For the 2D ablation experiments, only the 2D slice with the largest GTV tumor by surface area was used as input for both CT and PET. Everywhere else, the entire 3D scanning volume of the primary GTV was used.

The clinical data included age, sex, TNM classification stage as per the American Joint Committee on Cancer^48^—tumor size and invasion of surroundings, regional node involvment, and presence of metastasis—p16 biomarker status and primary tumor site. Sex and p16 biomarker status were encoded using categorical one-hot encoding representation. Similarly, primary tumour sizing (T) was encoded into values 0–4, regional lymph node spread (N) was encoded into values between 0 and 3 and distant metastasis (M) was encoded as 0 or 1. Age values were normalized between 0 and 1 based on a maximum value of 100 years. The resulting clinical data was a length 22 vector. Each endpoint (DM, LR, OS) was converted to a binary value for classification purposes. A ground-truth value designated the occurrence of the event for the patient at any point between the end of the treatment and the end of the follow-up period.

### PreSANet

**Figure 6:**
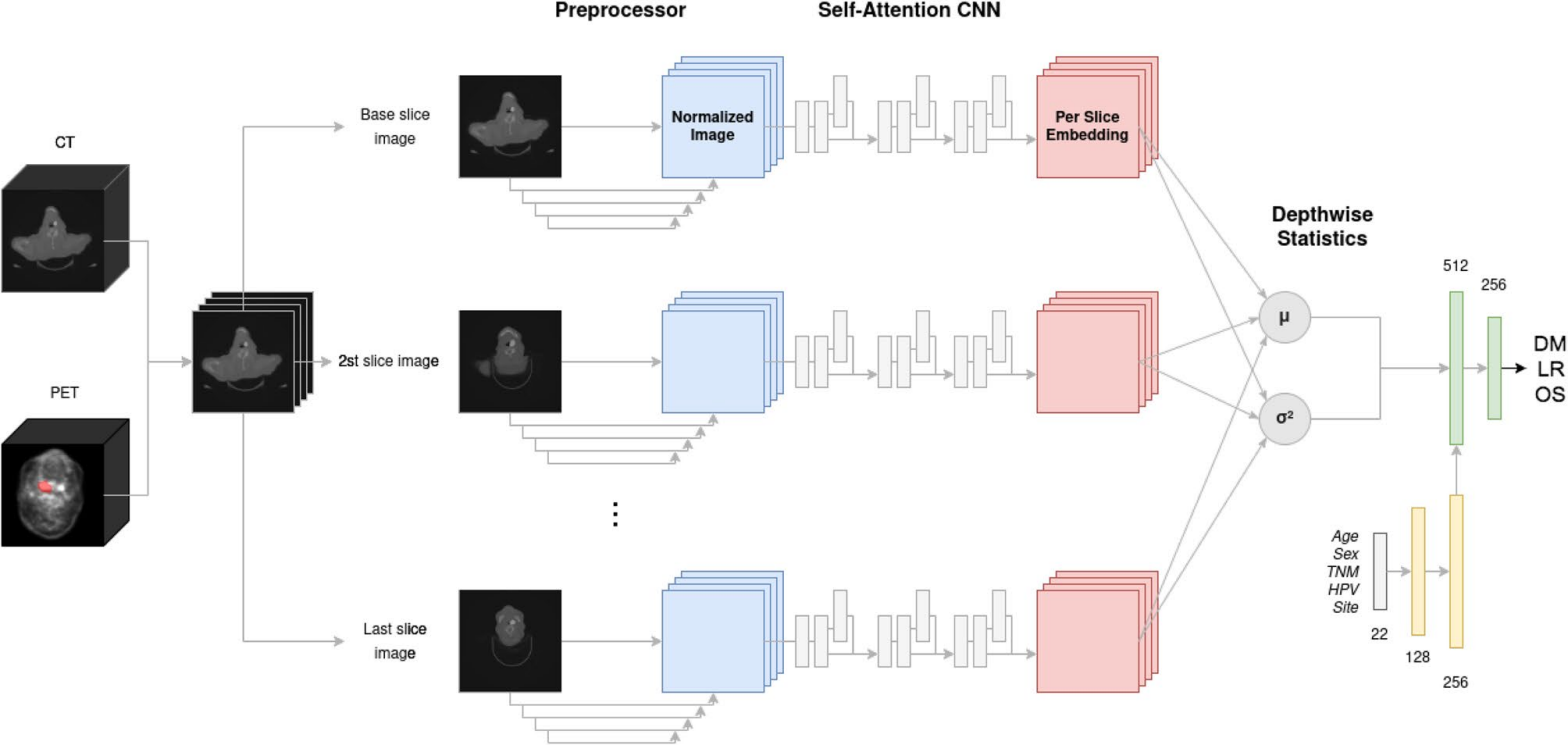
Proposed PreSANet deep learning architecture for radiotherapy outcome prediction. The convolutional backbones consist of the combination of the *preprocessor* module and the *self-attention* CNN feature extractor. Using shared weights, each of the two channel (PET, CT) input volume is split into individual slices passed to the backbone. The resulting per-slice feature vectors are aggregated using mean and variance into two 256-feature vectors. They are then concatenated with the outputs of a fully connected network component (yellow) that processes the clinical input data forming a 768 feature vector. Figure created with Draw.io

**Figure 7:**
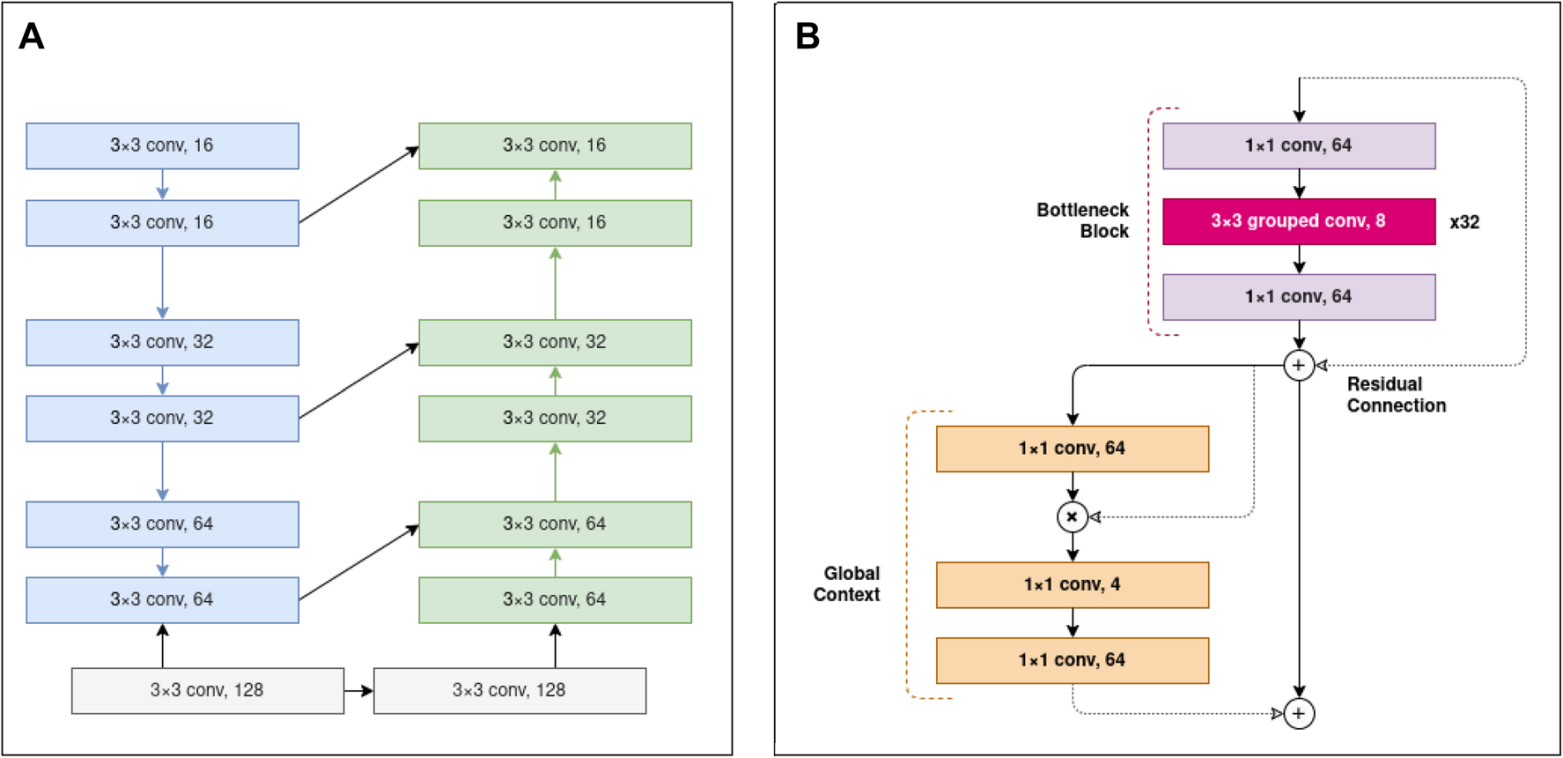
Details of the convolutional backbones of the proposed PreSANet model. (**A**) Preprocessor sub-module consisting of a downsampling and upsampling branch with skip connections taking a 2D image as input and returning a normalized 2D image as output. (**B**) Convolutional blocks in the feature extractor module consist of a residual bottleneck unit with depthwise-separable group convolutions followed by a global context unit. Every bottleneck unit outputs four times as many channels as it takes at its input, using 32 grouped convolutions with a $3\times 3$ kernel. Downsampling is performed in the middle layer of the bottleneck block using a stride of 2. Residual skip connections sum the input of the bottleneck block into its output. Self-attention is modelled with a context-modelling layer using an element-wise product. The residual addition of an attention map to the output of each block allows the model to process global context and attend salient parts of the image. Figure created with Draw.io

The proposed outcome prediction pipeline consists of an end-to-end pseudo-volumetric CNN with self-attention augmented with a dense auxiliary branch for clinical information integration, shown in Fig. 6. The first component of the model—the preprocessor module *Pre*—includes a low-capacity FCN network that performs image-to-image normalization as inspired by the works of Drozdzal et al.^34^. It consists of 4 downsampling convolutional layers with 16, 32, 64, and 128 filter maps, 3$\times$3 kernels and 2$\times$2 strides with a rectified linear unit (ReLU) activation layer after each, followed by 4 upsampling transpose convolutional layers with 128, 64, 32 and 16 filter maps, with matching kernel size, stride size and activation layers, shown in Fig. 7A. Between the layers with matching input/output size, long skip connections are used, implemented as channel-wise concatenation, to allow the model to spatially re-align the predicted normalized features. The resulting output is a deep normalized image, optimized with respect to the specific classification task.

Following this, the feature extraction component of the network is a CNN ($C_{SA}$), with 3 convolutional blocks with 64, 128 and 256 feature maps. Each convolutional block is comprised of a bottleneck triplet show in Fig. 7 with a 4 fold feature expansion factor: in the first block for example, a 1$\times$1 convolution layer with stride 1 and 64 feature maps is followed by a 32 depth wise-separable groups 3$\times$3 convolution layer—the bottleneck—with stride 2 (each group having 8 feature maps, totalling 256) that performs image downsampling, and finally by another 1$\times$1 convolutional layer with stride 1 and 64 feature maps. This approach helps to limit the size of the model in memory, often leading to better training performance^35^. Scaled exponential linear unit (SELU) activation function^49^ and batch-normalization are used after each convolution. Each convolutional block also includes a short residual skip connection around the bottleneck block to combat the exploding/vanishing gradient problem.

To benefit from the global contextual information in the latent image, a global context (GC) block with self-attention as presented by Cao et al.^36^ followed every convolution block. It was implemented as a 1$\times$1 convolutional layer and a softmax layer in the context modelling unit, two 1$\times$1 convolutional layers separated by layer normalization and ReLU activation inside the transformation block, and a final element-wise addition as fusion strategy with the residual input latent representation.

To integrate the patient clinical information into the CNN, an auxiliary branch $Aux^{(22)}(X_{clinical})$ was included taking as inputs the one-hot encoded categorical clinical features as well as the normalized age of the patient. This linear neural network consists of a 128 and a 256 dense layer, with SELU activation after each. The output feature vector was then concatenated to the flattened convolutional output vector.

In order to deal with the variability in GTV and cropped input volume size, we adapted the HeMIS^33^ model to combine information across all slices *x* in a volume *X*. This method normally combines any number of input modalities into a single features space by via an fusion layer, defined as the first and second moment of the hetero-modal inputs. In our case, the new volumetric abstraction layer is defined across heterogeneous input depths using the same two statistics for an input volume $X^{CT,PET}$ with two modalities as channels:

1 $$\begin{aligned}&\hat{E}\big [ X \big ] = \frac{1}{|X|} \sum _{x \in X} (C_{SA} \circ Pre)(x_{CT,PET}) \end{aligned}$$

2 $$\begin{aligned}&\hat{Var}\big [ X \big ] = \frac{1}{|X| - 1} \sum _{x \in X} \left[ (C_{SA} \circ Pre)(x_{CT,PET}) \right] ^{2} \end{aligned}$$

resulting in a single vector representing a patient’s 3D bi-modal (PET and CT) scan embedding. This pseudo-volumetric neural network approach for variable length inputs has the advantage of not necessitating any cropping and bounding-box artifacts that happens in more traditional volumetric methods. To do so, the entire convolutional backbone—Preprocessor *Pre* followed by the Self-Attention CNN $C_{SA}$—is used on each 2D input slice $x_{CT,PET} \in X$, with shared weights. The resulting per-slice representation is abstracted across all slices via the mean (Eq. 1) and variance (Eq. 2) statistics. The output yields two vectors of size 256, which is then concatenated together along with the clinical 256 feature vector to form a final 768-vector representation of the patient across variable scanning length, modalities and clinical data. This strategy is robust to missing slices, to interpolation artifacts and variable scan spacing, issues unique to medical imaging and uncommon to natural images, making it an attractive method for radiological scans. We compared this method with the standard approach of performing classification on a single slice containing the largest GTV lesion surface area^31^—a 2D approach.

To generate the final classification probability for each patient volume $X_{CT,PET}$ for a given target output *t*, a two hidden layer linear classifier neural network $L^{(768)}$ was used, with 128 and 256 features respectively, and each with a preceding SELU activation function layer to introduce non-linearity. This linear classifier takes as input the concatenation of the first moment $\hat{E}_{t}$, the second moment $\hat{Var}_{t}$ and the embedding of the clinical data $Aux^{(22)}_{t}$:

3 $$\begin{aligned}&P(X = t) = (Sigmoid \circ L^{(768)}_{t})(\hat{E}_{t}\big [ X_{CT,PET} \big ], \nonumber \\&\hat{Var}_{t}\big [ X_{CT,PET} \big ], Aux^{(22)}_{t}(X_{Clinical}) \text {\quad for \,} t \in \{DM, LR, OS\} \end{aligned}$$

where the resulting logits would then be used with a *Sigmoid* activation function to generate the per patient class probabilities $P(X = t)$ (Eq. 3). For training via stochastic gradient descent, the loss of the model is computed on these class probabilities via the binary cross-entropy.

### Network training

In total, each training fold was trained for 100 epochs on a single GeForce RTX 2080 Ti GPU with model checkpoints saved based on the top-1 validation accuracy. Training code and model architecture were implemented in Python 3.7.3^50^ and PyTorch 1.5.0^37^ using CUDA 10.1 and the PyTorch-Lightning^51^ library. To deal with data imbalance, a 6:1 resampling strategy was used on the minority class for each prediction label, along with online data augmentation by a factor of 4 using random flipping, shifts by up to $40\%$ and rotations by up to 20 degrees using the Albumentations^52^ library. The Adam optimizer was used with a learning rate of $3.2^{-5}$, a weight decay of $1.7^{-4}$ and a batch size of 32. Hyperparameters selection and model architecture choice was performed on the 20% validation subset of the training data and is presented in the ablation section of the results. The models were trained from scratch for each of the three possible outcome labels (DM, LR, OS), then fine-tuned via the proposed transfer learning strategy. For statistical power, the models were trained 5 times using different random initialization. Reported figures are the mean and $95\%$ confidence interval (CI) of the trials. Statistical significance was computed using the dependent t-test for paired samples and Spearman’s correlation for inter-fold comparison. Bonferroni corrections were applied for multiple comparisons to achieve the stated Type 1 error of 5%.

### Training and evaluation strategies

In each experiment performed in this study, transfer learning across prediction outputs was employed. Initially, each model was trained independently for each of the three target labels, DM, LR, and OS, resulting in three models. For each model, the state corresponding to the best validation performance (measured at each epoch of training) was kept. Then, of the three models, the one with the best validation performance was selected (and the others were discarded). Initializing the model weights with this model, transfer learning was performed by fine-tuning on the remaining two other labels.

**Figure 8:**
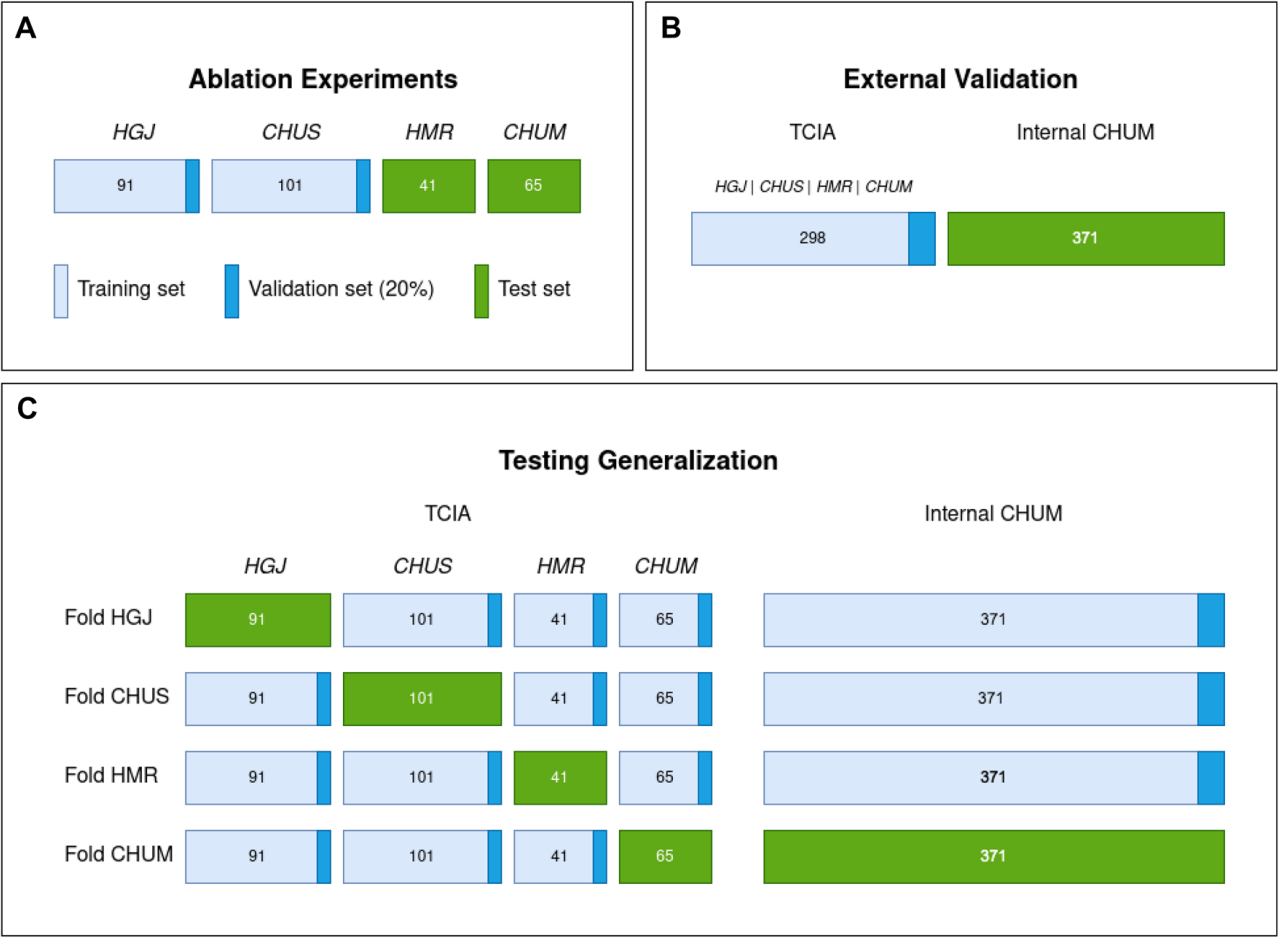
Data splitting strategies for model evaluation. The mean performance is reported across all five folds. (**A**) The training set are samples originating from CHUS and HGJ, with 20% held out as a validation set. The test set is composed of samples from CHUM and HMR. This split^30^ is used for ablation experiments as well as comparison to previous literature. (**B**) 5-fold cross-validation over the entire dataset. This split is used to evaluate the novel Internal CHUM dataset. (**C**) Hold-one-institution-out cross-validation strategy where each source institution is held as a test set with the remaining samples used for training and validation. This split is used to test cross-institution generalization. Figure created with Draw.io

Three separate data splitting strategies were evaluated, as shown in Fig. 8. These correspond to the four different phases of the study. Split A is used in two different phases: hyperparameters and model selection with ablation experiments, as well as comparison with literature and off-the-shelf models (Fig. 8A). Split B is used to evaluate the novel Internal CHUM dataset using the proposed model (Fig. 8B). Split C is used to evaluate the model generalizability to different sites using cross-validation (Fig. 8C).

In the first evaluation split (Fig. 8A), only patients from the TCIA “Head–Neck-PET–CT” dataset were considered. Samples from CHUS and HGJ—the two institutions forming the larger subsets with 192 (64%) samples—were selected to be part of the training and validation set, while the remaining are used for holdout testing. This split is identical to the one presented in the initial work in^30^ as well as in the follow-up work^31^. Also, the same split was used for the ablation experiments for hyperparameters and model selection, where a 20% subset of the training set was reserved as a validation set. This split is also used to compare model performance with results reported by the radiomics random forest approach by Vallières et al.^30^ and CNN by Diamant et al.^31^, as well as to compare with the following off-the-shelf computer vision models from the Torchvision package^53^: DenseNet^54^, InceptionV3^55^, ResNet^56^ and ResNeXt^35^ and a traditional survival prediction approach using Cox’s proportional hazards regression model based on the nomogram method^38^ (see Fig. S1 in appendix). This later method differs from all other machine learning methods presented as it only uses the clinical information. In this case, an additional outcome was trained for prediction called progression-free survival (PFS) which attempts at evaluating whether a patient would live without any form of recurrence or metastasis up to the last follow-up date. This prediction was generated as a combination of the individual prediction values of both the DM and the LR models, by taking the maximum score of the two individual probabilities.

In the second evaluation split (Fig. 8B), the model’s performance was evaluated on a separate external dataset, with training performed on the entire TCIA dataset. The Internal CHUM dataset was then used only for external validation: a 298:371 training testing split. This split is useful as a true holdout testing on an unseen dataset. It also has the particularity that the testing split contains samples from CHUM, which is an institution seen during training in the TCIA dataset. Overall this particular split considers the effects of training on a multi-site dataset on performance generalization without enforcing any multi-domain restriction.

In the third evaluation strategy shown in Fig. 8C, we investigate if withholding one of the domains from the training set affects model performance. In other words, we explore the generalization power of the proposed model to new unseen domains rather than unseen datasets (which was explored in split B). Specifically, for each hospital (CHUM, CHUS, HGJ and HMR), samples from that site were reserved for the testing set: the rest of the data was used for training and validation. The overall methodology is functionally a non-randomized 4-fold cross-validation. In this way, performance can be evaluated on testing data from an unseen site, with the training set benefiting from features from three other sites. This would allow demonstrating whether the proposed model was robust to variation across acquisitions from different institutions.

Performance evaluation was repeated five times for every model in all of the results to obtain confidence intervals and ensure accurate statistical power. The monitored metrics included the area under the Receiver Characteristic Curve (AUROC), accuracy, specificity and sensitivity for the binary classification task. Accuracy figures are balanced for each class due to the imbalanced nature of the dataset.

### Ethical statement

This study was performed in line with the principles of the Declaration of Helsinki. Ethics approval was granted by the Institutional Review Board (IRB) for human studies of the Centre Hospitalier Université de Montréal (CHUM). Informed consent was waived by the IRB (Comité Éthique de la Recherche 18.194) of CHUM as the data used in the study was retrospective.

## Supplementary Information

Supplementary Information.

## References

[1] Delaney G, Jacob S, Featherstone C, Barton M (2005). The role of radiotherapy in cancer treatment: Estimating optimal utilization from a review of evidence-based clinical guidelines. Cancer Interdiscip. Int. J. Am. Cancer Soc..

[2] Begg AC, Stewart FA, Vens C (2011). Strategies to improve radiotherapy with targeted drugs. Nat. Rev. Cancer.

[3] Barton MB, Delaney GP (2011). A decade of investment in radiotherapy in new south wales: Why does the gap between optimal and actual persist?. J. Med. Imaging Radiat. Oncol..

[4] Atun R (2015). Expanding global access to radiotherapy. Lancet Oncol..

[5] Kayalibay, B., Jensen, G. & van der Smagt, P. CNN-based segmentation of medical imaging data. *arXiv preprint* arXiv:1701.03056 (2017).

[6] Lee J-G (2017). Deep learning in medical imaging: General overview. Korean J. Radiol..

[7] Erickson BJ, Korfiatis P, Akkus Z, Kline TL (2017). Machine learning for medical imaging. Radiographics.

[8] Mohan G, Subashini MM (2018). MRI based medical image analysis: Survey on brain tumor grade classification. Biomed. Signal Process. Control.

[9] Mohsen H, El-Dahshan E-SA, El-Horbaty E-SM, Salem A-BM (2018). Classification using deep learning neural networks for brain tumors. Future Comput. Inform. J..

[10] Sahiner B (2019). Deep learning in medical imaging and radiation therapy. Med. Phys..

[11] Razzak, M. I., Naz, S. & Zaib, A. Deep learning for medical image processing: Overview, challenges and the future. In *Classification in BioApps*, 323–350 (Springer, 2018).

[12] Afshar, P., Mohammadi, A. & Plataniotis, K. N. Brain tumor type classification via capsule networks. In *2018 25th IEEE International Conference on Image Processing (ICIP)*, 3129–3133 (IEEE, 2018).

[13] Giger ML (2018). Machine learning in medical imaging. J. Am. Coll. Radiol..

[14] Yeh S-A (2010). Radiotherapy for head and neck cancer. Semin. Plast. Surg..

[15] Devlin, J., Chang, M., Lee, K. & Toutanova, K. BERT: pre-training of deep bidirectional transformers for language understanding. *CoRR*. arxiv:1810.04805 (2018).

[16] Radford, A. *et al.* Language models are unsupervised multitask learners (2019).

[17] Voulodimos A, Doulamis N, Doulamis A, Protopapadakis E (2018). Deep learning for computer vision: A brief review. Comput. Intell. Neurosci..

[18] Guo Y (2016). Deep learning for visual understanding: A review. Neurocomputing.

[19] Oprea, S. *et al.* A review on deep learning techniques for video prediction. arxiv:2004.05214 (2020). 10.1109/TPAMI.2020.3045007 33320810

[20] Fawaz HI, Forestier G, Weber J, Idoumghar L, Muller P-A (2019). Deep learning for time series classification: A review. Data Min. Knowl. Discov..

[21] Shen D, Wu G, Suk H-I (2017). Deep learning in medical image analysis. Annu. Rev. Biomed. Eng..

[22] Garcia-Garcia, A., Orts-Escolano, S., Oprea, S., Villena-Martinez, V. & Garcia-Rodriguez, J. A review on deep learning techniques applied to semantic segmentation. *arXiv preprint* arXiv:1704.06857 (2017).

[23] Viergever, M. A. *et al.* A survey of medical image registration—Under review (2016). 10.1016/j.media.2016.06.030 27427472

[24] Wang T (2021). A review on medical imaging synthesis using deep learning and its clinical applications. J. Appl. Clin. Med. Phys..

[25] Litjens G (2017). A survey on deep learning in medical image analysis. Med. Image Anal..

[26] Zhang Q, Yang LT, Chen Z, Li P (2018). A survey on deep learning for big data. Inf. Fusion.

[27] Deng, J. *et al.* ImageNet: a large-scale hierarchical image database. In *CVPR09* (2009).

[28] Vallieres, M. *et al.* Data from head-neck-pet-CT. The cancer imaging archive (2017).

[29] Clark K (2013). The cancer imaging archive (TCIA): Maintaining and operating a public information repository. J. Digit. Imaging.

[30] Vallières M (2017). Radiomics strategies for risk assessment of tumour failure in head-and-neck cancer. Sci. Rep..

[31] Diamant A, Chatterjee A, Vallières M, Shenouda G, Seuntjens J (2019). Deep learning in head & neck cancer outcome prediction. Sci. Rep..

[32] Rawat W, Wang Z (2017). Deep convolutional neural networks for image classification: A comprehensive review. Neural Comput..

[33] Havaei, M., Guizard, N., Chapados, N. & Bengio, Y. Hemis: Hetero-modal image segmentation. *CoRR*. arxiv:1607.05194 (2016).

[34] Drozdzal M (2018). Learning normalized inputs for iterative estimation in medical image segmentation. Med. Image Anal..

[35] Xie, S., Girshick, R. B., Dollár, P., Tu, Z. & He, K. Aggregated residual transformations for deep neural networks. *CoRR*. arxiv:1611.05431 (2016).

[36] Cao, Y., Xu, J., Lin, S., Wei, F. & Hu, H. Gcnet: Non-local networks meet squeeze-excitation networks and beyond. *CoRR*. arxiv:1904.11492 (2019).

[37] Paszke A, Wallach H (2019). Pytorch: An imperative style, high-performance deep learning library. Advances in Neural Information Processing Systems.

[38] Ju J (2016). Nomograms predicting long-term overall survival and cancer-specific survival in head and neck squamous cell carcinoma patients. Oncotarget.

[39] McKinney SM (2020). International evaluation of an AI system for breast cancer screening. Nature.

[40] Li Y (2019). Post-radiotherapy PET/CT for predicting treatment outcomes in head and neck cancer after postoperative radiotherapy. Eur. J. Nucl. Med. Mol. Imaging.

[41] Tanaka H (2021). Circulating tumor HPV DNA complements PET-CT in guiding management after radiotherapy in HPV-related squamous cell carcinoma of the head and neck. Int. J. Cancer.

[42] Robin P (2015). Diagnostic performance of FDG PET/CT to detect subclinical HNSCC recurrence 6 months after the end of treatment. Eur. J. Nucl. Med. Mol. Imaging.

[43] Fahey F (2010). Variability in pet quantitation within a multicenter consortium. Med. Phys..

[44] Kops, E., Schmitz, T. & Herzog, H. Geometric accuracy of reconstructed pet images. In *1998 IEEE Nuclear Science Symposium Conference Record. 1998 IEEE Nuclear Science Symposium and Medical Imaging Conference (Cat. No.98CH36255)*, vol. 3, 1904–1906 (1998).

[45] Shahinfar S, Meek P, Falzon G (2020). How many images do i need? Understanding how sample size per class affects deep learning model performance metrics for balanced designs in autonomous wildlife monitoring. Ecol. Inform..

[46] Narayana PA (2020). Deep-learning-based neural tissue segmentation of MRI in multiple sclerosis: Effect of training set size. J. Magn. Reson. Imaging.

[47] Barbedo JGA (2018). Impact of dataset size and variety on the effectiveness of deep learning and transfer learning for plant disease classification. Comput. Electron. Agric..

[48] Edge SB, Compton CC (2010). The American joint committee on cancer: the 7th edition of the AJCC cancer staging manual and the future of TNM. Ann. Surg. Oncol..

[49] Klambauer, G., Unterthiner, T., Mayr, A. & Hochreiter, S. Self-normalizing neural networks. *CoRR*. arxiv:1706.02515 (2017).

[50] Van Rossum G, Drake FL (2009). Python 3 Reference Manual.

[51] Falcon, W. Pytorch lightning. *GitHub*. Note: https://github.com/williamFalcon/pytorch-lightning Cited by **3** (2019).

[52] Buslaev, A. *et al.* Albumentations: Fast and flexible image augmentations. *Information* **11** (2020). https://www.mdpi.com/2078-2489/11/2/125.

[53] Marcel, S. & Rodriguez, Y. Torchvision the machine-vision package of torch. In *Proceedings of the 18th ACM International Conference on Multimedia, MM ’10*, 1485–1488 (Association for Computing Machinery, New York, NY, USA, 2010). 10.1145/1873951.1874254.

[54] Huang, G., Liu, Z., Van Der Maaten, L. & Weinberger, K. Q. Densely connected convolutional networks. In *Proceedings of the IEEE conference on computer vision and pattern recognition*, 4700–4708 (2017).

[55] Szegedy, C. *et al.* Going deeper with convolutions. In *Proceedings of the IEEE Conference on Computer Vision and Pattern Recognition*, 1–9 (2015).

[56] He, K., Zhang, X., Ren, S. & Sun, J. Deep residual learning for image recognition. In *Proceedings of the IEEE Conference on Computer Vision and Pattern Recognition*, 770–778 (2016).

