# Supplementary material for: Cross-institutional outcome prediction for head and neck cancer patients using self-attention neural networks
Source: Sci Rep. 2022 Feb 24;12:3183. doi: 10.1038/s41598-022-07034-5 (PMC8873259; doi:10.1038/s41598-022-07034-5)
Supplement: Supplementary file 1 — Supplementary Information. [file 41598_2022_7034_MOESM1_ESM.pdf]

Appendix

Table S1. Model performance on the Internal CHUM dataset using a randomized 5-fold cross-validation. Comparison between CT+clinical and PET+CT+clinical input modalities. Clinical data is included in all cases. Accuracy is normalized per class frequency.

Input	Metric	DM	LR	OS
CT+clinical	Accuracy	**68.9 [68.6-69.3]**	**74.1 [67.6-80.6]**	**74.2 [67.7-80.5]**
	Specificity	**85.3 [80.8-89.9]**	**73.1 [58.9-88.2]**	**72.9 [60.2-84.3]**
	Sensitivity	**52.5 [47.6-57.4]**	75.0 [65.9-85.3]	**75.0 [64.0-85.8]**
	AUROC	**65.3 [62.1-68.4]**	**68.3 [58.1-78.6]**	**68.2 [63.5-73.0]**
PET+CT+clinical	Accuracy	65.2 [58.1-72.4]	66.8 [61.8-71.9]	62.4 [58.7-66.2]
	Specificity	82.8 [71.2-94.4]	58.3 [40.2-76.4]	57.6 [31.7-83.5]
	Sensitivity	47.8 [28.9-66.7]	**75.4 [61.5-89.4]**	67.3 [43.3-91.4]
	AUROC	64.7 [50.6-78.7]	58.8 [47.9-69.7]	55.4 [48.5-62.2]

Table S2. Ablation performance of models using all possible input modalities. Figures are *Mean [95 % Confidence Interval]* over 5 trials with different seeds. AUROC is computed on the Receiver operating characteristic curve. Statistical significance testing is computed using the Mann-Whitney U test with Bonferroni correction against the proposed model. Stars indicate statistical significance using the dependent t-test for paired samples, with the number of stars indicating: 1) $p < 0.05$, 2) $p < 0.01$.

	3D	Preprocessor	Self-Attention	AUROC	Statistical Significance
DM	✓	✓	✓	**79.8 [77.0–82.7]**	—
DM	✓		✓	66.2 [61.5–71.0]	⋆
DM	✓	✓		63.5 [58.8–68.3]	⋆
DM	✓			63.2 [61.5–65.0]	⋆
DM		✓	✓	60.6 [55.7–65.5]	⋆
DM			✓	62.9 [59.2–66.2]	⋆
DM		✓		62.8 [56.1–69.5]	⋆
DM				65.7 [62.6–68.8]	⋆
LR	✓	✓	✓	**78.8 [77.0–80.5]**	—
LR	✓		✓	63.8 [56.1–71.5]	⋆
LR	✓	✓		65.0 [61.1–69.0]	⋆
LR	✓			70.5 [64.3–76.5]	
LR		✓	✓	66.8 [60.8–72.9]	⋆
LR			✓	70.3 [63.5–77.2]	
LR		✓		68.2 [60.8–75.6]	⋆
LR				68.8 [67.1–70.4]	⋆
OS	✓	✓	✓	**82.0 [80.2–83.9]**	—
OS	✓		✓	77.4 [74.5–80.2]	
OS	✓	✓		78.8 [76.0–81.6]	
OS	✓			82.2 [81.0–83.3]	
OS		✓	✓	72.6 [67.0–78.2]	⋆
OS			✓	75.4 [73.3–77.5]	⋆
OS		✓		77.6 [76.7–78.4]	⋆
OS				75.7 [72.4–79.0]	⋆

Table S3. Ablation performance of pseudo-3D models on samples from HMR and CHUM. Figures are means over 5 trials with different seeds. AUROC is computed on the Receiver operating characteristic curve. Accuracy is balanced for class distribution.

Label	Preprocessor	Attention	CT	clinical	PET	AUROC (%)	CI (95%)	Accuracy (%)	Specificity (%)	Sensitivity (%)
DM	✓	✓	✓	✓	✓	**79.8**	[77.0–82.7]	**74.5**	**84.9**	64.2
DM	✓	✓	✓		✓	60.6	[52.3–68.9]	60.6	55.4	65.7
DM	✓	✓	✓	✓		65.1	[62.9–67.4]	68.1	64.8	71.4
DM	✓	✓	✓			57.0	[47.1–66.9]	63.8	53.3	74.3
DM		✓	✓	✓	✓	66.2	[61.5–71.0]	65.0	70.0	60.0
DM		✓	✓		✓	59.1	[51.0–67.2]	63.1	59.1	67.1
DM		✓	✓	✓		64.8	[63.3–66.4]	68.0	60.2	75.7
DM		✓	✓			66.8	[64.8–68.8]	67.2	64.4	70.0
DM	✓		✓	✓	✓	63.5	[58.8–68.3]	65.4	66.5	64.3
DM	✓		✓		✓	52.8	[45.5–60.0]	57.4	30.4	**84.3**
DM	✓		✓	✓		66.7	[63.1–70.4]	69.1	61.1	77.1
DM	✓		✓			54.0	[46.9–61.1]	58.8	46.1	71.4
DM			✓	✓	✓	63.2	[61.5–65.0]	64.4	60.2	68.6
DM			✓		✓	47.9	[43.22–52.6]	56.6	54.6	58.6
DM			✓	✓		67.4	[65.1–69.7]	65.8	68.7	62.9
DM			✓			54.3	[51.5–57.2]	55.8	53.0	58.6
LR	✓	✓	✓	✓	✓	**78.8**	[77.0–80.5]	**74.2**	81.0	67.5
LR	✓	✓	✓		✓	59.3	[52.5–66.2]	64.2	48.4	80.0
LR	✓	✓	✓	✓		66.0	[63.7–68.4]	63.8	77.6	50.0
LR	✓	✓	✓			61.0	[53.0–69.0]	62.8	49.3	76.3
LR		✓	✓	✓	✓	63.8	[56.1–71.5]	65.1	65.1	65.0
LR		✓	✓		✓	71.7	[67.4–75.9]	68.3	74.0	62.5
LR		✓	✓	✓		74.6	[68.9–80.3]	70.6	62.4	78.8
LR		✓	✓			66.9	[60.9–72.8]	68.5	64.4	72.5
LR	✓		✓	✓	✓	65.0	[61.1–69.0]	64.9	66.0	63.8
LR	✓		✓		✓	64.4	[57.7–71.2]	64.9	57.3	72.5
LR	✓		✓	✓		67.0	[64.8–69.1]	68.4	74.2	62.5
LR	✓		✓			65.9	[61.3–70.4]	64.3	53.6	75.0
LR			✓	✓	✓	70.4	[64.3–76.5]	68.1	77.3	58.8
LR			✓		✓	59.9	[52.4–67.4]	61.8	41.1	**82.5**
LR			✓	✓		68.4	[62.0–74.7]	68.0	**82.2**	53.8
LR			✓			67.0	[64.7–69.2]	64.8	57.1	72.5
OS	✓	✓	✓	✓	✓	82.0	[80.2–83.9]	78.7	80.7	76.7
OS	✓	✓	✓		✓	56.5	[51.5–61.4]	57.3	58.8	55.8
OS	✓	✓	✓	✓		82.0	[79.7–84.3]	**79.5**	77.3	81.7
OS	✓	✓	✓			68.5	[65.6–71.5]	66.3	57.6	75.0
OS		✓	✓	✓	✓	77.4	[74.5–80.2]	73.6	71.5	75.8
OS		✓	✓		✓	59.2	[53.6–64.9]	56.5	50.5	62.5
OS		✓	✓	✓		80.7	[77.9–83.5]	78.2	72.2	**84.2**
OS		✓	✓			72.2	[70.0–74.4]	71.8	59.5	**84.2**
OS	✓		✓	✓	✓	78.8	[76.0–81.6]	76.0	**82.0**	70.0
OS	✓		✓		✓	59.5	[52.4–66.6]	60.0	43.4	76.7
OS	✓		✓	✓		80.5	[77.9–83.2]	77.7	75.4	80.0
OS	✓		✓			58.7	[48.2–69.1]	61.7	50.0	73.3
OS			✓	✓	✓	82.2	[81.0–83.3]	79.8	80.5	79.2
OS			✓		✓	70.3	[69.0–71.6]	66.7	61.7	71.7
OS			✓	✓		**83.7**	[83.2–84.2]	78.3	78.3	78.3
OS			✓			71.8	[69.7–73.9]	71.6	61.5	81.7

Table S4. Ablation performance of 2D models on samples from HMR and CHUM. Figures are means over 5 trials with different seeds. AUROC is computed on the Receiver operating characteristic curve. Accuracy is balanced for class distribution.

Label	Preprocessor	Attention	CT	clinical	PET	AUROC (%)	CI (95%)	Accuracy (%)	Specificity (%)	Sensitivity (%)
DM	✓	✓	✓	✓	✓	60.6	[56.0–65.5]	59.3	54.7	63.9
DM	✓	✓	✓		✓	64.6	[58.8–70.5]	62.9	61.3	64.5
DM	✓	✓	✓	✓		68.3	[66.6–70.1]	63.2	72.5	53.9
DM	✓	✓	✓			67.0	[64.2–69.7]	62.0	65.1	58.9
DM		✓	✓	✓	✓	62.7	[59.2–66.2]	61.3	51.1	71.5
DM		✓	✓		✓	66.8	[66.0–67.6]	62.5	**78.6**	46.5
DM		✓	✓	✓		66.9	[60.4–73.4]	63.6	71.8	55.4
DM		✓	✓			75.2	[73.7–76.7]	71.9	68.7	75.1
DM	✓		✓	✓	✓	62.8	[56.1–69.5]	58.8	67.2	50.4
DM	✓		✓		✓	69.5	[66.7–72.3]	64.5	70.7	58.3
DM	✓		✓	✓		69.5	[66.7-72.4]	57.4	30.4	84.3
DM	✓		✓			**83.8**	[82.5–85.0]	**80.3**	72.1	88.5
DM			✓	✓	✓	65.7	[62.6–68.8]	62.5	65.8	59.1
DM			✓		✓	54.9	[52.5–57.2]	51.3	52.7	**99.9**
DM			✓	✓		66.1	[62.8–69.5]	61.0	57.0	64.9
DM			✓			65.5	[64.9–66.1]	64.3	63.3	65.3
LR	✓	✓	✓	✓	✓	66.8	[60.8–72.9]	62.3	66.1	58.6
LR	✓	✓	✓		✓	59.2	[53.5–64.9]	63.2	70.1	56.3
LR	✓	✓	✓	✓		64.0	[59.0–69.0]	63.6	87.4	39.9
LR	✓	✓	✓			66.6	[60.2–73.0]	63.7	86.2	41.2
LR		✓	✓	✓	✓	70.3	[63.5–77.2]	65.1	63.7	66.4
LR		✓	✓		✓	69.3	[62.0–76.5]	64.9	86.8	42.9
LR		✓	✓	✓		65.1	[63.0–67.1]	64.4	86.0	42.8
LR		✓	✓			55.4	[47.4–63.3]	57.2	86.2	28.3
LR	✓		✓	✓	✓	68.2	[60.8–75.6]	64.8	72.6	56.9
LR	✓		✓		✓	62.6	[55.7–69.6	63.2	86.6	39.8
LR	✓		✓	✓		64.4	[57.7–71.2]	64.9	57.3	**72.5**
LR	✓		✓			57.9	[55.8–60.0]	57.0	76.9	37.2
LR			✓	✓	✓	68.8	[67.1–70.4]	63.8	83.0	44.5
LR			✓		✓	61.3	[60.0–62.5]	61.0	70.6	51.5
LR			✓	✓		**73.1**	[68.8–77.3]	**66.4**	92.1	40.8
LR			✓			51.6	[51.3–51.8]	50.0	**100.0**	0.0
OS	✓	✓	✓	✓	✓	72.6	[67.0–78.2]	66.3	65.8	66.8
OS	✓	✓	✓		✓	73.4	[71.9–74.8]	68.2	65.2	71.2
OS	✓	✓	✓	✓		74.2	[70.4–77.9]	**73.1**	**82.9**	63.4
OS	✓	✓	✓			76.7	[74.8–78.6]	70.8	69.1	72.4
OS		✓	✓	✓	✓	75.4	[73.3–77.5]	68.4	62.7	74.2
OS		✓	✓		✓	74.8	[71.0–78.6]	72.1	77.5	66.8
OS		✓	✓	✓		75.4	[73.2–77.7]	69.2	65.4	73.0
OS		✓	✓			73.1	[71.1–75.1]	69.4	66.9	71.8
OS	✓		✓	✓	✓	**77.6**	[76.7–78.4]	66.7	56.9	76.6
OS	✓		✓		✓	76.6	[74.5-78.7]	71.9	75.5	68.2
OS	✓		✓	✓		74.9	[70.9–79.0]	73.0	79.9	66.0
OS	✓		✓			75.0	[71.9–78.1]	70.1	76.2	64.0
OS			✓	✓	✓	75.7	[72.4–79.0]	67.6	60.5	74.7
OS			✓		✓	59.3	[56.3–62.3]	50.0	0.0	**100.0**
OS			✓	✓		75.4	[70.5–80.2]	68.8	62.5	75.1
OS			✓			62.1	[61.6–62.6]	50.0	0.0	**100.0**

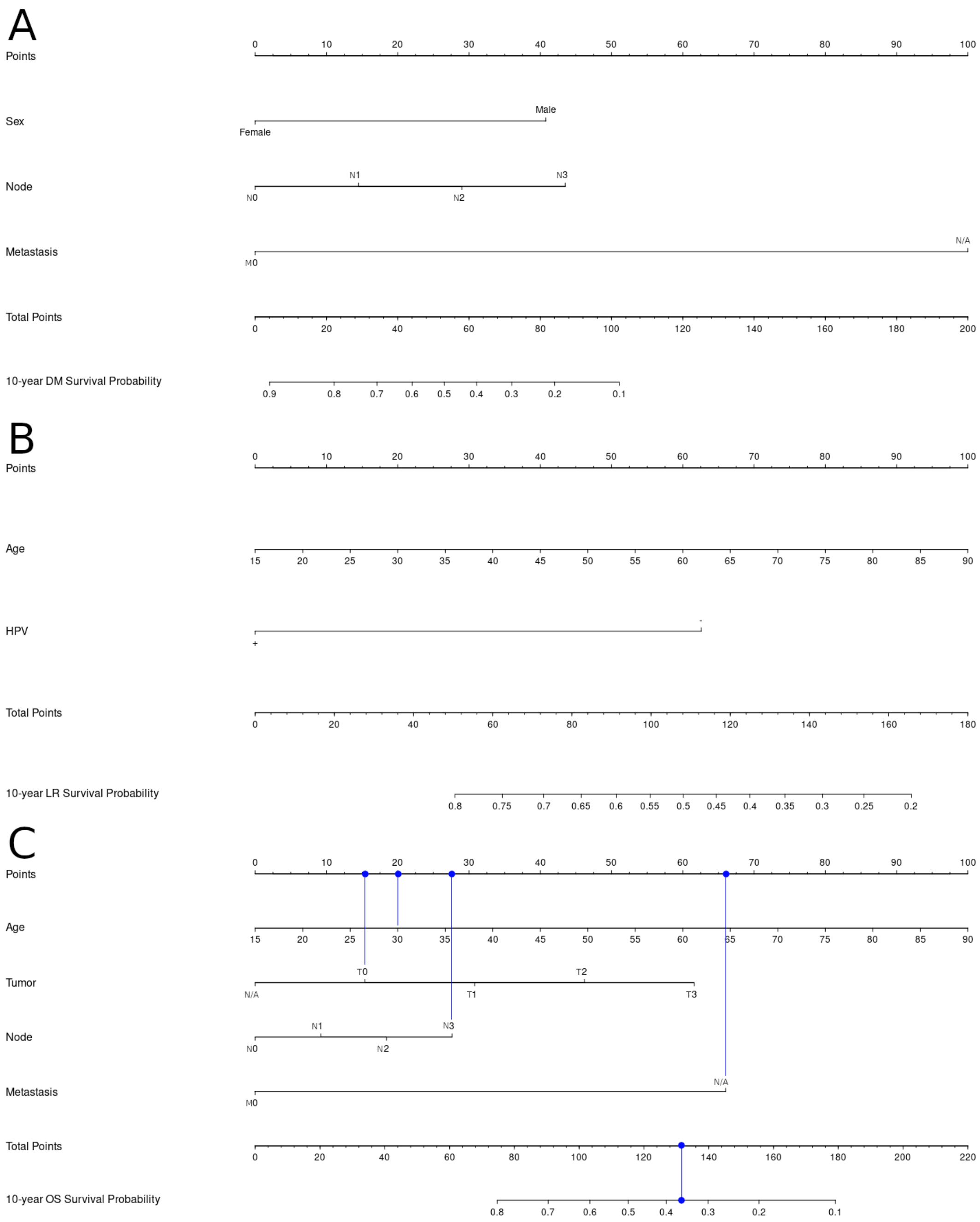

Figure S1. Nomograms predicting 10 years survival outcome for the predicted events: A) DM, B) LR, C) OS. The model used was a Cox's Proportional Hazard model[38] trained on the TCIA dataset's clinical factors. The included model features were optimized by minimizing the Akaike Information Criterion. The training samples included only those from CHUS and HGJ institutions. **To read**: draw a vertical line from each factor to the Points line and perpendicular to both (factor and points lines), calculate the total sum of points and draw a line from that total sum value on the Total Points line, to the 10-year survival line. The probability for the outcome is where the line crosses the 10-year survival probability via an intersection line, also perpendicular to both total points and 10-year survival probability lines. **Example** (blue vertical lines, C): patient with *age=30* (20 points), and scores for *tumor=T0* (15 points), *node=N3* (27.5 points) and *metastasis=unknown* (66 points). The total sum of 128.5 points gives a score of 36% chance of survival after 10 years for OS. Figure created with the "rms" package v6.2 (cran.r-project.org/web/packages/rms).
